# Using stochastic epidemiological models to evaluate conservation strategies for endangered amphibians

**DOI:** 10.1098/rsif.2017.0480

**Published:** 2017-08-30

**Authors:** Brian Drawert, Marc Griesemer, Linda R. Petzold, Cheryl J. Briggs

**Affiliations:** 1 Department of Computer Science, University of North Carolina Asheville, Asheville, NC 28804, USA; 2 Biosciences and Biotechnology Division, Lawrence Livermore National Laboratory, Livermore, CA 94551, USA; 3 Department of Computer Science, University of California, Santa Barbara, CA 93106, USA; 4 Department of Ecology, Evolution and Marine Biology, University of California, Santa Barbara, CA 93106, USA

**Keywords:** chytridiomycosis, *Batrachochytrium dendrobatidis*, epidemiological models, stochastic models

## Abstract

Recent outbreaks of chytridiomycosis, the disease of amphibians caused by the fungal pathogen *Batrachochytrium dendrobatidis* (Bd), have contributed to population declines of numerous amphibian species worldwide. The devastating impacts of this disease have led researchers to attempt drastic conservation measures to prevent further extinctions and loss of biodiversity. The conservation measures can be labour-intensive or expensive, and in many cases have been unsuccessful. We developed a mathematical model of Bd outbreaks that includes the effects of demographic stochasticity and within-host fungal load dynamics. We investigated the impacts of one-time treatment conservation strategies during the disease outbreak that occurs following the initial arrival of Bd into a previously uninfected frog population. We found that for all versions of the model, for a large fraction of parameter space, none of the one-time treatment strategies are effective at preventing disease-induced extinction of the amphibian population. Of the strategies considered, treating frogs with antifungal agents to reduce their fungal load had the greatest likelihood of a beneficial outcome and the lowest risk of decreasing the persistence of the frog population, suggesting that this disease mitigation strategy should be prioritized over disinfecting the environment or reducing host density.

## 1. Introduction

Amphibian populations around the world are being severely impacted by the disease chytridiomycosis, caused by the chytrid fungus *Batrachochytrium dendrobatidis* (commonly referred to as ‘Bd’) [1,2]. Disease outbreaks following the initial invasion of the fungus into naive amphibian populations can result in massive mortality events, frequently leading to extreme population declines or local extinction of the amphibian host populations. For example, a wave of chytridiomycosis swept through high elevation areas of Central America [3,4], contributing to the extirpation of many species in previously diverse amphibian communities. In the California Sierra Nevada, chytridiomycosis outbreaks have nearly eliminated a previously widespread and abundant, but now endangered, frog species complex (mountain yellow-legged frogs; *Rana muscosa* and *Rana sierra*) from hundreds of mountain lakes [5,6].

The recent rapid declines in amphibian biodiversity are unprecedented [7], although chytridiomycosis is only one of a number of contributing factors [8]. It has been suggested that ‘it is morally irresponsible to document amphibian declines and extinction without also designing and promoting a response to this global crisis’ [9]. One *ex situ* conservation strategy that is currently being implemented is the development of captive-assurance colonies (e.g. the Amphibian Ark [10]), in which individuals of highly threatened amphibian species have been brought into captivity, with the hope that they can be released at a later point in time. This *ex situ* approach is justified in part by the fact that there is currently no well-proven method for mitigating the impacts of chytridiomycosis in the wild, although this is an area of a great deal of active research [11–13]. Many efforts are underway to develop disease mitigation strategies that can be implemented in amphibian populations in their natural environment, although to date there has been only limited success in field trials of these strategies [14–16].

Bd has been able to invade amphibian populations in some of the most remote areas of the world; therefore, any strategy that involves attempting to prevent the spread of Bd into previously uninfected sites is unlikely to be successful in the long term. For example, Bd has recently been found in Madagascar, which was previously thought to be Bd-free [17].

Mathematical models have frequently served as a useful tool to guide mitigation efforts against human and wildlife diseases [18–22]. In this paper, we use mathematical models to help inform and guide conservation efforts to prevent chytridiomycosis-induced extinction of amphibian populations. Specifically, we investigate whether any conservation strategies can change the outcome of a chytridiomycosis outbreak from extirpation of the amphibian hosts to persistence of the host population. Our goals are to (i) elucidate mechanisms by which conservation strategies are most likely to be effective at increasing the fraction of frogs that survive through the initial chytridiomycosis outbreak and (ii) assess the efficacy and potential risks that these strategies have on the host amphibian populations. We focus here on the effectiveness of three types of one-time treatment strategies: treating frogs with antifungal agents, reducing the density of the host population and treating the environment with antifungals to reduce the pool of infectious zoospores.

## 2. Background

The amphibian chytrid fungus Bd is transmitted via a motile, aquatic zoospore. The zoospore encysts on keratinized tissues of amphibian hosts (which occur in the mouthparts of tadpoles, and in the skin of post-metamorphic stages), and develops a single sporangium (also called zoosporangium, the structure in which spores are formed). New zoospores (motile spore that uses a flagellum for locomotion) develop in the sporangium and are released to the environment [23,24], although Bd can sometimes also spread from cell to cell within amphibian skin [25]. Under laboratory conditions, zoospores generally remain motile for only a short period (from a few hours to a few days, depending on the temperature [26–28]); however, there is the potential for Bd to persist outside of amphibian hosts, although the typical duration and mechanisms of environmental persistence are still under investigation [29–31]. Bd is a generalist pathogen, with the capacity to infect most amphibian species (and also, potentially, a number of non-amphibian species [32–34]). The outcome of infection with Bd, however, varies greatly between amphibian species, with adults of some species succumbing rapidly to chytridiomycosis and others showing high levels of tolerance to infection with the fungus [1,2]. In susceptible species, chytridiomycosis can kill hosts through disrupting osmoregulation and normal skin function [35,36]. In a number of species, infected individuals are not impacted by low-level infections (with low fungal loads) but die when fungal loads are high [37].

Our analysis is based on a system with a single host species, the mountain yellow-legged frogs complex: *Rana muscosa* and *Rana sierrae* (henceforth *R. muscosa*). *Rana muscosa* are native to California's Sierra Nevada mountains, including Yosemite, Kings Canyon and Sequoia National Parks. They are highly aquatic, typically inhabiting lakes, ponds, marshes, meadows and streams at elevations ranging from 4500 to 12 000 feet. This species has experienced significant population declines and local extinctions over the last four decades [37], and was listed as ‘endangered’ under the US Endangered Species Act in 2014 [38]. One advantage of using this species for our analysis is that due to the remote habitat, it is an ideal system to study the host–pathogen dynamics in relative isolation; thereby reducing the complexity of interactions in multi-species systems.

### 2.1. Potential conservation strategies

We investigate the efficacy of three general classes of conservation strategies that involve single treatments applied at discrete points in time. For simplicity, in our analysis, we examined each of these conservation strategies independently rather than in combination. We did not assume any details of their particular implementation (e.g. we did not assume that a particular antifungal drug was used), rather we varied the timing and fraction of either the host population or environment treated. Field implementations of these strategies might seek to combine treatments together for maximum effect.

#### 2.1.1. Clearing individuals of infection

A number of methods are available for clearing tadpoles and/or post-metamorphic individuals of Bd infection, or at least reducing their fungal loads. These include anti-fungal agents, such as drugs normally used to treat fungal infections in humans, e.g. itraconazole [12,39–43] or voriconazole [44], fungicides normally used to target plant pathogens (e.g. thiophanate-methyl [45]), antibiotics (e.g. chloramphenicol [46]) and even sodium chloride [43]. Additionally, elevated temperatures can clear Bd infections on individuals of amphibian species that can tolerate the thermal stress [47–50]. Many of these approaches have been used successfully to clear individuals of infection in laboratory experiments and captive assurance colonies; however, their use in field trials has been limited. One recent field trial showed promising results [16].

#### 2.1.2. Reducing host density

In many host–pathogen models, reducing the density of susceptible hosts through culling can limit the severity of disease outbreaks [51,52]. The mechanism by which reduction of host density can lead to positive outcomes is typically due to a density-dependent transmission of the pathogen. If the density is reduced below a critical threshold, the disease will typically fade out [53]. Culling has at times been successful at eliminating diseases in domestic animal populations [21,54], especially when the cull concentrates on eliminating all infected host individuals. However, it has rarely been successful at controlling disease in populations of wild animals [55,56], and recent models suggest that it is unlikely to be successful at controlling emerging infectious diseases in other wildlife species (e.g. facial tumours in Tasmanian devils [57] and white nose syndrome in bats [58]). Culling has never been seriously considered as a strategy for control of amphibian chytridiomycosis in natural populations; however, reducing the density of susceptible hosts in a population through capture and relocation of a fraction of the infected population is a strategy that has been implemented [59].

#### 2.1.3. Disinfecting the environment

Many of the same antifungal agents that have been used to treat individuals can also potentially be used to disinfect the environment in which the amphibians live. Disinfection of the environment may be difficult to implement in complex environments; however, it has been successful in small, isolated ponds in Mallorca, where researchers eliminated Bd by completely draining, drying out and refilling the ponds [60].

#### 2.1.4. Other conservation treatment strategies

In addition to the one-time treatment strategies described above, there is a large class of intervention strategies that involve permanent alterations to the host–pathogen system. A sensitivity analysis of this class of conservation measures can be found in electronic supplementary material, §4.1. One notable example of this type of strategy is the application of probiotic bacteria applied to the skin of amphibians [10,61–64]. However, changes to the bacterial community may be complex and evolving, and requires a more detailed model to understand the potential benefits and risks of this type of treatment.

## 3. Models

The model is inspired by the system observed in the Sierra Nevada in which *R. muscosa* is often the only amphibian species present, thus we consider only a single host species. Our model describes the dynamics of the initial arrival of Bd into an uninfected frog population, and the subsequent disease outbreak. Because we focus on the time scale of the initial epizootic, we ignore births and deaths by other causes. In the Sierra Nevada systems, all adults can succumb to chytridiomycosis within a single season (one to three months), while the time to maturation for *R. muscosa* is multiple years [6].

The dynamics of the fungal load (i.e. the number of sporangia *S*_*i*_) on each individual frog *i* = 1 ….*N* (where *N* is the number of live frogs), and the population of Bd zoospores *Z* in the pool, are described by3.1
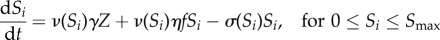
and3.2
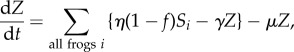
where frog *i* dies for *S*_*i*_ > *S*_max_. The parameters are defined in table 1. We assume that the system is spatially homogeneous and well mixed. In some variants of the model (described below), *σ* or *ν* can depend on the individual's current fungal load.

**Table 1.** Model parameters and ranges of their values. All parameters were sampled on a linear scale except for *γ*, which was sampled on a logarithmic scale. *Z* represents zoospores, *S* represents sporangia.

parameter	range	units	description
*N*_frogs_	5–200	frogs	initial number of frogs
*γ*	10^−6^ to 10^0^	frog^−1^d^−1^	zoospore encounter rate
*η*	5–20	*Z* *S*^−1^d^−1^	zoospore production rate
*ν*_0_	0–1	*S* *Z*^−1^	zoospore encystment rate
*f*	0–1	dimensionless	zoospore host reinfection fraction
*σ*_0_	0.1–0.5	d^−1^	sporangia shedding rate
*μ*	0.01–1.5	d^−1^	zoospore death rate
*S*_max_	10 000	S	lethal threshold for sporangia load

Because many amphibian species are not impacted by low-level Bd infections but succumb to the disease chytridiomycosis at high Bd fungal loads [6,65], determining the potential for intervention strategies to control specific Bd outbreaks requires an understanding of the dynamics of the fungal load on individual hosts. We, therefore, investigated three versions of a model that make different assumptions about what (if anything) can regulate the fungal load on a host individual. The models are based on the individual-based model of Briggs *et al.* [37], which envisages a population of frogs sharing a common waterbody (a lake or pond).

**Figure 1.**
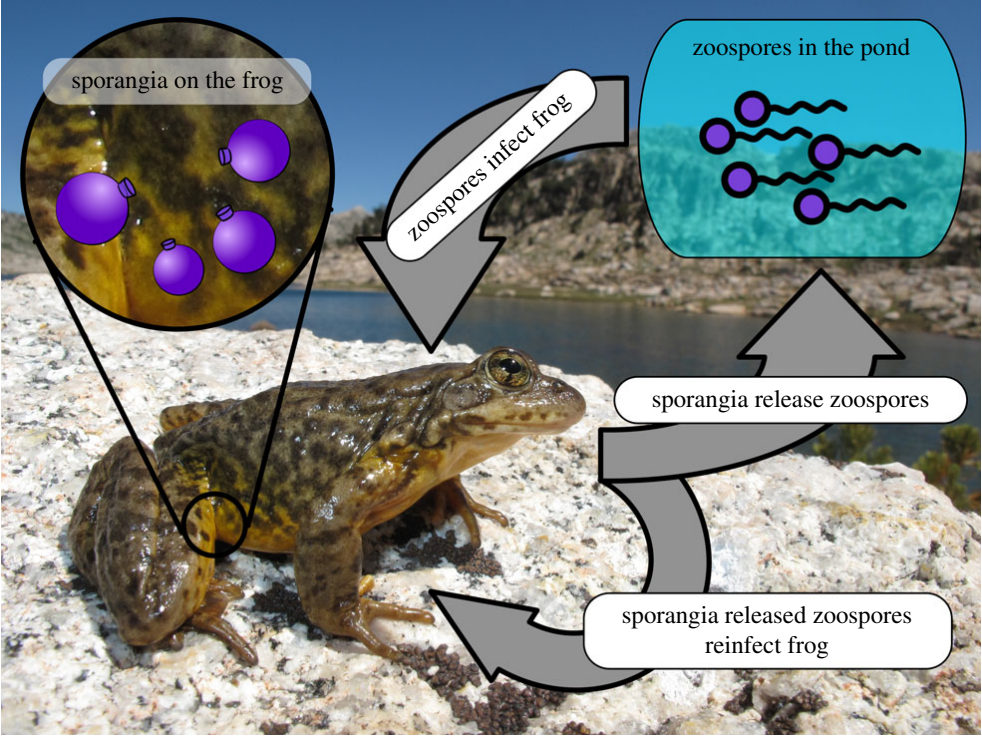
Diagram showing the life cycle of *Batrachochytrium dendrobatidis*. Motile zoospores in the pond encounter frogs and encyst in the stratum granulosum layers of frog skin, forming sporangia. The sporangia mature and release zoospores into the environment. Released zoospores can immediately reinfect the host frog, thereby increasing its fungal load, or be released into the pond to infect other frogs in the population. Photo credit: Devin Edmonds, USGS.

The model assumes that the Bd load on a frog can increase through both encountering zoospores in the zoospore pool and reinfection from zoospores produced by sporangia on its own skin (figure 1). The transmission rate from zoospores in the pool to frog *i* is given by *ν*(*S*_*i*_)*γ*, where *γ* is the encounter rate between frogs and zoospores, and *ν*(*S*_*i*_) is the fraction of these encounters that result in successful encystment of the zoospore on frog *i*. Once on the skin, sporangia generate zoospores at a rate *η*. The model assumes that a fraction *f* of the released zoospores immediately re-encounter the same host (and a fraction *ν* successfully encyst), while the remaining fraction (1 − *f*) of the released zoospores enter the zoospore pool. Sporangia on the skin of frog *i* die at a rate *σ*(*S*_*i*_) (due to sloughing of frog skin and other causes), and free-swimming zoospores in the zoospore pool die at rate *μ*. Table 1 describes these parameters and their ranges of values. In the model, individual frogs are unaffected by low-level Bd infections, but a frog dies when its Bd load *S*_*i*_ exceeds a threshold *S*_max_, as has been observed for a number of amphibian species [6,65]. When a frog dies, the model assumes that all sporangia on that frog also die (in the field, Bd loads are found to decline rapidly after frog death [66]).

### 3.1. Load-dependent model variants

We investigated three variants of the model that make different assumptions about how the Bd load on a host varies as the disease progresses. (i) The Baseline version, which assumes constant values for both *ν*(*S*_*i*_) = *ν*_0_ and *σ*(*S*_*i*_) = *σ*_0_, was based on observations on Bd in the Sierra Nevada, in which Bd loads on frogs grow exponentially during epidemics [6] (note that constant rate results in exponential growth, see electronic supplementary material, §1.1). However, it has been observed in some amphibian systems that the Bd growth rate on individuals initially increases exponentially, but then levels off at higher loads [67]. This is followed, in some cases, by a decline in load [68]. This type of Bd load dynamics on individual frogs requires modification to the model to allow for decreased Bd growth rate at high loads. To accommodate this, we investigated two additional model variants: (ii) a version where the zoospore encystment rate decreases with load (the Nu model) 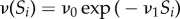 and (iii) a version where the sporangia shedding rate increases with load (the Sigma model) *σ*(*S*_*i*_) = *σ*_0_ + *σ*_1_*S*_*i*_.

### 3.2. Parametrization and non-dimensionalization

Although exact empirical estimates for most of the parameters in the model are not available, all of the parameters have specific biological interpretations that allow us to constrain their values to realistic ranges (table 1). Many of the parameters vary among habitats or among amphibian species, so we performed a global sensitivity analysis across the entire realistic ranges of the parameters. We constrained our choices of parameter combinations to include only those for which there is an outbreak of chytridiomycosis leading to death of all frogs within 14–275 days [6] in the deterministic version of the Baseline model, in the absence of any disease intervention strategy.

A number of the parameters in our model are not uniquely identifiable in terms of their effects on the dynamics of the model. Therefore, in order to efficiently cover the parameter space in our global sensitivity analysis, we first recast the system of equations into a non-dimensional form [69] (table 2, where the description of each parameter is also given).

**Table 2.** Non-dimensional parameters and their descriptions.

parameter	definition	description
c1	*ην fσ*^−1^	self-reinfection rate
c2	*γη*(1 − *f*)*ν N*_frogs_*σ*^−2^	infectiousness of the frogs
c3	*γN*_frogs_ *σ*^−1^	zoospore loss rate due to interacting with the frogs
c4	*μσ*^−1^	zoospore background loss rate

We used a Monte Carlo rejection method to sample the parameter space, to ensure that points were not too close together in parameter space (described in the electronic supplementary material, §1.3). The result was 2132 unique points which sufficiently cover our parameter space. The Nu and Sigma models each have 4264 parameter points. The total number of parameter points for all models without conservation was 10 660.

### 3.3. Implementation of conservation strategies

We implemented the three one-time conservation strategies described above: cleaning, culling and cleaning the environment. For each of these, we explored the effect of a one-time application of the treatment on our suite of models, along two parameter axes: day of intervention and fraction of the (frog or zoospore) population treated. The first parameter axis is the timing of the application of the conservation strategy relative to the invasion of Bd into the population and the onset of Bd infection. We explored applying the conservation strategies at 14, 28 and 56 days after the initial infection. The second parameter axis is the fraction of the frog or zoospore population treated in the conservation method. We explored the application of the method at 25%, 50% and 75% effectiveness (fraction of the population that is subjected to the treatment). In total, we attempted 27 different conservation strategies on each of the 10 660 parameter points, yielding a total number of points within our parameter space of 298 480.

### 3.4. Simulation methods

We ran simulations to examine the efficacy of the various intervention strategies during the epidemic that occurs following the invasion of Bd into a previously naive population. In all simulations, we introduce a single infected frog with an initial Bd load of *S*_1_(0) = 100 sporangia into an otherwise uninfected population of *N*_frogs_ (*S*_*i*_(0) = 0 for *i* = 2 … *N*), in a pond initially containing no zoospores (*Z*(0) = 0).

We investigated both deterministic and stochastic versions of each of the three variants of the model. For the deterministic (ordinary differential equation (ODE)) version, we obtained numerical solutions using a 4th/5th order Runge–Kutta algorithm (Gnu Scientific Library [70]). For the stochastic version of the model, we implemented a variant of the Gillespie [71] stochastic simulation algorithm, treating frogs, zoospores and sporangia as discrete (integer) entities, and treating the rates as probabilities of discrete events (transmission, death, etc.), see electronic supplementary material, §1.3. The state variables for the stochastic system are the number *N* of frogs alive in the pond, the number *Z* of zoospores in the pond and the sporangia load *S*_*i*_ on each frog *i*. We ran 30 stochastic trajectories for each combination of parameters, and computed statistics (mean and standard deviation) for our analysis. To facilitate reproduction of these results, we have included all of our simulation source code as the electronic supplementary material.

## 4. Results

**Figure 2.**
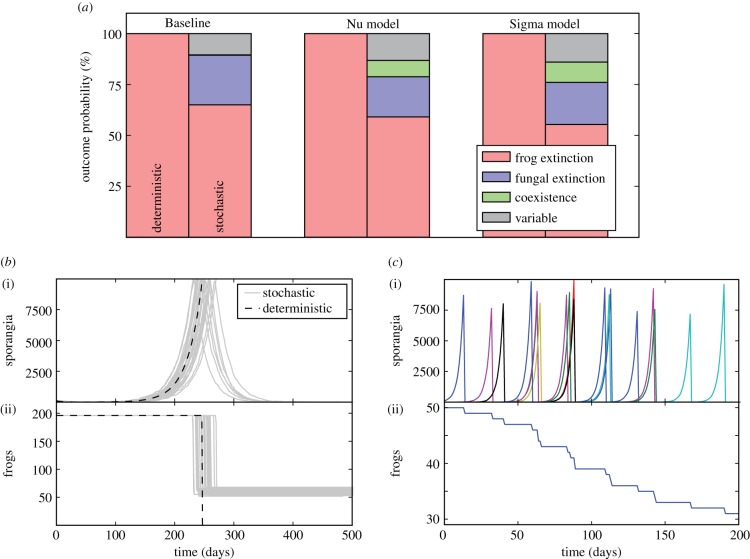
Difference between stochastic and deterministic outcomes without conservation. (*a*) Deterministic outcome for the Baseline model is always extinction—by construction. Stochastic simulation of the same parameter set reveals a variety of outcomes. The ‘variable’ outcome means that different stochastic realizations of the model give different outcomes. (*b*) Thirty trajectories (of the same model) show that stochasticity can lead to population survival for the Baseline model. (i) The number of sporangia on the initially infected frog (black: deterministic trajectory; grey: stochastic trajectories). The distribution in the time of death for the frogs, due to the randomness of the growth rate of the sporangia load, results in a fraction of the population dying and the remainder living due to a decrease in the pathogen density. This leads to fungal die-out and amphibian survival. (*c*) A single trajectory illustrating the ‘stuttering chain’ effect, where only a small number of frogs are infected at any one time, transmitting the pathogen to uninfected frogs. (i) The fungal load on each frog in the population, where each colour represents the sporangia on a different frog. As the frog population diminishes, the probability of successful transmission decreases until the pathogen goes extinct.

Stochasticity plays an important role in determining the outcome of invasion of Bd into naive frog populations. The parameter values were deliberately selected such that outbreaks of chytridiomycosis would invariably occur if any intervention strategy was not implemented, resulting in frog population extinctions for the deterministic formulations of all variants of the model (Baseline, Nu and Sigma) without intervention. This allowed us to explore the efficacy of the various intervention strategies, because in the deterministic formulations of the models, anything other than complete frog extinction represents a positive intervention outcome. However, the stochastic formulation of the model can produce a variety of different outcomes for this same set of parameters, even in the absence of any interventions (figure 2*a*). For example, for the Baseline model without interventions, frog extinction occurs in 100% of the cases (points in parameter space) in the deterministic formulation, but only in about 65% of the cases for the stochastic formulation, with fungal extinction occurring in 24% of the cases, and the frogs and fungus coexisting in about 0.01% of the cases. In the remaining approximately 10% of the cases, the outcome is variable. This means that, for the same parameters, more than one outcome is observed for different stochastic realizations of the model, i.e. the frogs being driven extinct in some runs and the fungus going extinct in other runs. Similar differences in the outcomes between the deterministic and stochastic formulations are seen for the Nu and Sigma models.

The differences between the deterministic and stochastic formulations of the model stem from two phenomena, which we refer to as ‘partial die-offs’ and ‘stuttering chains’, which occur is different part of parameter space. The ‘partial die-offs’ phenomenon results from the fact that the stochastic trajectories produce a distribution of times until death of the frogs in a population, while in the deterministic version of the model, the fungal load dynamics are identical on all frogs (except for the single initially infected frog). Coupled with the density-dependent nature of the disease, this can lead to the phenomenon illustrated in figure 2*b*, in which the disease outbreak results in only a ‘partial die-off’ of the frog population. As the frogs begin to die due to the disease, the frog population drops below the critical density for positive growth of the fungus before the fungal loads on all frogs reach their lethal threshold, *S*_max_. This results in a subsequent loss of fungal infection from the remaining frogs. The ‘stuttering chains’ phenomenon occurs in the cases where the growth rate of the fungal infection on the frog is large, but the transmission rate to other frogs is relatively low. In these cases, we see a ‘stuttering chain’ [72] of transmission, in which only a small number of frogs in the population are infected at one time. Because each infected frog has a decreasing probability of transmitting the infection to another frog as the population size diminishes, the chain of infected individuals ends before the population is driven extinct. This process is illustrated in figure 2*c*.

**Figure 3.**
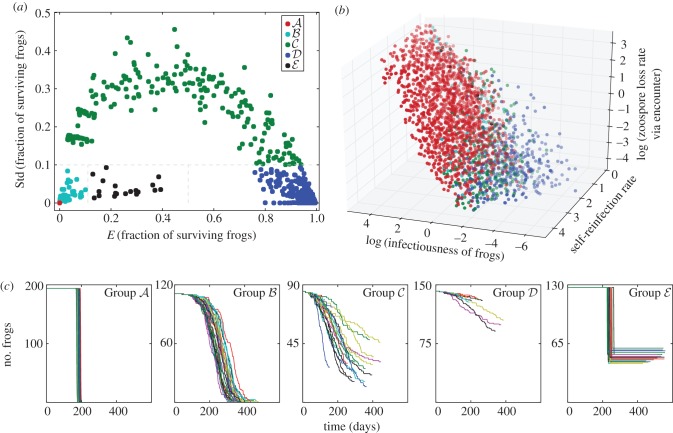
Regions of differing dynamical behaviour. (*a*) Division of the dynamical space based on the mean and standard deviation of the fraction of surviving frogs into five subgroups. Grey dashed lines show the boundaries. Group 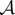 (red) is defined as the points with mean and standard deviation of zero. Group 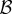 (teal) points have mean and standard deviation both less than 0.1. Group 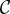 (green) points have standard deviation greater than 0.1. Group 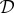 (blue) points have mean greater than 0.5 and standard deviation less than 0.1. Group 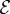 (black) points have mean greater than 0.1 and less than 0.5, with a standard deviation less than 0.1. (*b*) A scatter plot showing the location of the subgroups within a three-dimensional slice of the non-dimensional parameter space. Note that, for much of parameter space (the points in Group 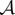), the outcome is extinction. The non-dimensional parameter *c*1 is the self-reinfection rate, *c*2 is the infectiousness of the frogs and *c*3 is the zoospore loss rate. (*c*) Simulated trajectories from one representative parameter point in each of the five groups. Each point is chosen to be representative of the dynamics in that group. Each panel shows 30 stochastic trajectories of the frog population over the time course of the outbreak. Trajectories that end at values greater than zero frogs indicate fungal extinction.

We found it informative to divide our parameter space into five groups based on the mean and standard deviation of the fraction of frogs surviving across the 30 stochastic realizations for each parameter combination (shown for the stochastic Baseline model in figure 3). The points in Group 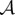 (red) represent the 65% of parameter combinations that behave like the deterministic version, with 100% frog extinction for all stochastic runs. At the other end of the spectrum, the points in Group 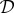 (blue) correspond to parameter combinations for which the pathogen always fails to invade and the frog population always persists. For points in this group, a few frogs in the population may get infected and die in a ‘stuttering chain’, but the pathogen invariably fades out prior to infecting a substantial fraction of the population. The parameter combinations along the arc in figure 3*a* fall into Groups 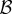 (cyan) and 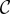 (green), which represent a continuum between Groups 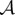 and 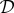, where stochastic effects dominate the chain of transmission, and different fractions of the frogs become infected prior to fade-out of the pathogen (figure 3*a*). The points in Group 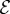 (black) represent parameter combinations for which stochasticity leads to the ‘partial die-off’ phenomenon. For these points, a positive growth rate of the Bd load on individual frogs requires continues infection from the zoospore pool. Therefore, the death of a fraction of the population reduces the frog density and thus reduces the force of infection, allowing the remaining frogs to lose their infection and survive (figure 3*c*).

Figure 3*b* shows the colour-coded location in parameter space for three axes of the non-dimensionalized parameters (see electronic supplementary material, figure S9, for a more detailed illustration). We see that the self-reinfection rate (*c*1), infectiousness of frogs (*c*2) and the zoospore loss rate via encounters with frogs (*c*3) all have some influence on the specific group to which each parameter point is assigned, while zoospore background loss rate (*c*4, not shown) did not significantly predict the outcome. Overall, we found that infectiousness of frogs (*c*2) is most predictive of survival outcome. There is a *c*2 transition, below which there is high survival, above which there is no survival and a band in the middle for which the outcome is variable. These results agree with the results from our statistical analysis, which can be found in electronic supplementary material, §2.

### 4.1. Effectiveness of conservation measures

**Figure 4.**
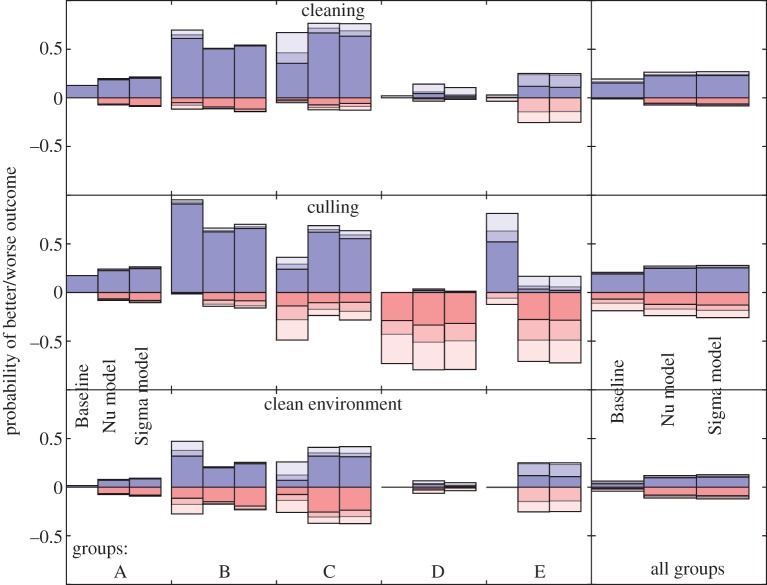
Effectiveness of each of the conservation methods. Left shows effectiveness by group (figure 3); right shows effectiveness across all groups. Blue bars show the fraction of parameter points for which the application of the conservation measure improves the mean fraction of frogs surviving by at least 10%/30%/50% (light/medium/dark), respectively. Red bars show the fraction of points where the outcome is decreased by at least 10%/30%/50% (light/medium/dark), respectively. The sign (+/ −) of the *y*-axis indicates whether the conservation strategy had a positive or negative effect.

The outcomes of the one-time conservation measures are shown in figure 4. Here, an outcome is considered to be positive (or alternatively, negative) if the fraction of frogs surviving increased (decreased) by at least 10%, 30% or 50% (as indicated by light/medium/dark shading) due to the conservation measure, in comparison to taking no action. The fraction of the parameter space that had a positive (negative) conservation outcome is shown by the relative size of the blue (red) bar. The fraction that had neither an increase nor decrease is not shown. The analogous data for the deterministic model are shown in electronic supplementary material, figure S13.

Looking at our conservation methods over all groups (right column of figure 4), we see that cleaning frogs has a positive effect in 20–30% of the parameter space, and rarely has a negative effect. Culling frogs can have a greater chance of positive effect (compared with cleaning frogs), but has an equally large chance of being detrimental to the frog population (because frogs are removed in the process). Thus, culling is a much riskier conservation method. Cleaning the environment is largely ineffective, as it has only a small chance of any effect and both positive and negative outcomes are equally likely.

We also looked at the effects of the conservation methods for each of our parameter groups. We found that Group 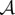 sees very little effect from the conservation methods. This mirrors what we see in the deterministic version of the model. For the parameters in Group 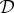, little is going to improve the survival of the frog populations as they already have a high survival fraction in the absence of conservation measures. Thus, we see that cleaning has little effect and culling has a large negative effect as it is killing (or removing) animals that would have otherwise survived. The groups that show the greatest impact from conservation strategies are Groups 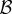 and 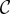, the ones with both high stochasticity and high mortality. In these groups, cleaning has a large probability of a positive effect and a small chance of a negative effect. Culling has a large probability of a positive effect, but also a significant chance of a negative effect. This is due to the fact that these populations are very likely to be driven extinct without any intervention, and both cleaning and culling may be able to break the chain of infection. Cleaning of the environment has small and equal probabilities of a positive or negative effect.

The dynamics of Group 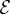 tend to exhibit the ‘partial die-off’ phenomena. Thus, both cleaning of the frogs and cleaning of the environment have little effect. For the Baseline model, culling has a high probably of a positive effect, due to the effect of reducing the frog density on the density-dependent dynamics. For the Nu and Sigma models, a large fraction of frogs survive for parameters in Group 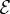 without any intervention, thus culling has a high probability of a negative effect.

For the deterministic version of the model, electronic supplementary material, figure S13 illustrates that most of the interventions are relatively ineffective. In particular, none of the interventions lead to a positive outcome for the deterministic Baseline model, and cleaning of the environment never has a positive effect for any of the variants of the deterministic model. Cleaning the frogs also has only a very small chance of a positive effect. The one group that benefits greatly from conservation is the culling of Group 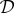. In the stochastic version, this group has a high survival rate in the absence of intervention, but only extinction in the deterministic version. Culling in the deterministic version changes the density dynamics of the disease, causing the Bd growth rate to be driven negative, resulting in disease clearance and frog survival.

The effectiveness of each of the conservation methods, broken down by the day and fraction of the population treated, is shown in electronic supplementary material, figure S14. For cleaning, treating a larger fraction always increased the probability of a positive outcome. In general, cleaning earlier (14 days after pathogen introduction) is more likely to yield a positive outcome than cleaning later (56 days). For culling, increasing the fraction culled or culling that fraction early in the outbreak, not only increases the likelihood of a positive outcome, but also increases the likelihood of a negative outcome. The effectiveness of cleaning the environment is relatively insensitive to both timing and fraction of the zoospore pool removed. These trends are similar for all three variants of the model.

## 5. Discussion

While a number of treatment options are available that can eliminate Bd infections on individual amphibians, or reduce the density of infectious zoospores in the environment [11], implementing these mitigation strategies under field conditions can be costly and labour-intensive. Additionally, it is unlikely that every individual in an amphibian population can be captured and treated, or that Bd zoospores can be completely eliminated from the habitat. Here, we focus exclusively on the situation in which Bd arrives in a naive population of amphibians, and would lead to a disease outbreak and amphibian extinction if no intervention strategies are implemented.

We investigated intervention strategies that involved one-time treatments that are applied as a pulse perturbation to the system. Of the treatment options considered, we found that using any of the available antifungal protocols (e.g. itraconazole treatments [40]) to clear the infection from at least a fraction of the frogs is the most likely to have a beneficial outcome, and is relatively unlikely to reduce amphibian population persistence. Culling can sometimes be effective at allowing some frogs in the population to survive the Bd outbreak, but this strategy is more risky than treating frogs with antifungal agents, because (by definition) it reduces the size of the host population. Although reducing the density of hosts can sometimes push the population below the threshold for disease invasion or persistence [53] (assuming the system has density-dependent transmission), reducing the size of a population can also increase its risk of extinction due to other causes (e.g. demographic and environmental stochasticity, random catastrophes and genetic drift) [73,74]. One-time antifungal treatments applied to the environment to remove infectious Bd zoospores are unlikely to have a negative effect on the fraction of frogs that survive a Bd outbreak, however. Our models suggest that this type of approach is also relatively unlikely to reduce the impact of Bd on the amphibian population, at least during the epizootic phase of disease dynamics. Additionally, we note that none of the available antifungal compounds are specific to Bd; therefore, widespread use in the environment may have additional, non-target detrimental effects.

One surprising outcome from the model was the lack of sensitivity to the parameter controlling the background death rate of zoospores (as shown by low sensitivity of *μ* or *c*4 in electronic supplementary material, tables S7 and S8, and electronic supplementary material, figures S7, S8, S11 and S12). Thus, although conservation strategies aimed at reducing zoospore survival in the environment (e.g. via addition of predators of zoospores, such as the zooplankton, *Daphnia*) have received a great deal of recent attention in the literature [75–77], our models suggest that they may be relatively ineffective at preventing disease-induced amphibian extinction, at least during the epizootic phase of the disease dynamics. Predators of zoospores may, however, play an important role in determining the pathogen prevalence and infection intensity if Bd persists with the host in a long-term enzootic state [77]. Two more complex conservation strategies that we do not fully explore with our simple model are those that alter the resistance or tolerance of the host through either the stimulation of the host's immune response, or the augmentation of the community of beneficial bacteria.

We also investigated a class of intervention strategies that involve permanent alterations to the system (see electronic supplementary material, §4.1); we found that the pathogen growth rate is most sensitive to the zoospore encystment success *ν*, the zoospore release rate *η* and the lifespan of sporangia on the host *σ*^−1^. Strategies that can effectively decrease these parameters have potential for reducing the impacts of Bd outbreaks. This suggests that the recent attention devoted to the development of probiotic bacteria applied to the skin of amphibians [10,61–64] is warranted, if bacteria taxa can be found that can persist on the amphibian skin and provide a long-term reduction in susceptibility to this fungal pathogen (i.e. effectively reducing *ν*).

In 2013, a second species of *Batrachochytrium*, *B. salamandrivorans* (Bsal), was identified and is resulting in similar outbreaks in salamander species in Europe and is leading to die-offs of salamanders in this region [78,79]. There is concern about this pathogen spreading from its current areas of infection to hot spots of salamander biodiversity, such as regions of North America [80]. The lessons learned from conservation efforts in the fight against Bd will be critical in informing disease mitigation strategies against Bsal [81]. Strategies that combat this initial stage of invasion are precisely what is needed in the event that Bsal continues its spread [82] and invades North American salamander populations. Our modelling framework may also be applied to the control of other fungal diseases of wildlife [83]. Therefore, the scenario investigated in our model (mitigation efforts aimed at reducing the impact of the initial invasion of the pathogen into a naive host population) will be highly relevant to the control of Bsal if and when it invades North America.

## Supplementary Material

Supplementary Information Text

## Supplementary Material

Simulation Code

## Data accessibility

The simulation code and parameter data have been uploaded as part of the electronic supplementary material.
