# Supplementary material for: Using stochastic epidemiological models to evaluate conservation strategies for endangered amphibians
Source: J R Soc Interface. 2017 Aug 30;14(133):20170480. doi: 10.1098/rsif.2017.0480 (PMC5582134; doi:10.1098/rsif.2017.0480)
Supplement: Supplementary Information Text [file rsif20170480supp1.pdf]

Supplementary Information
for
Using Stochastic Epidemiological Models to Evaluate Conservation Strategies for Endangered Amphibians
published in
Journal of the Royal Society Interface

Brian Drawert, Marc Griesemer, Linda Petzold, and Cheryl J. Briggs

1 Epidemiological Model of Frog-Bd Dynamics

The deterministic equations for the dynamics of the fungal load (the number of sporangia S_i) on each individual frog i, and the population of Bd zoospores Z in the pool are given by

$$\frac{dS_i}{dt} = \gamma\nu(S_i)Z + \nu(S_i)\eta f S_i - \sigma(S_i)S_i \qquad \text{for} \qquad S_i \leq S_{max} \tag{1}$$

$$\frac{dZ}{dt} = \sum_{all\, frogs\, i} \{\eta\,(1-f)\,S_i - \gamma Z\} - \mu Z. \tag{2}$$

For the deterministic version, we obtained numerical solutions to equations (1-3) using a 4th/5th order Runge-Kutta algorithm (part of the Gnu Scientific Library[1]).

1.1 Model Variants

1.1.1 Baseline Model: $\nu(S_i) = \nu_0$, $\sigma_i = \sigma_0$

Our Baseline Model follows the assumptions of the Briggs et al. (2010) [2] model, in which both the fraction of zoospores that successfully encyst upon encountering a host, and the death rate of sporangia, are set to constant values: $\nu(S_i) = \nu_0$ and $\sigma(S_i) = \sigma_0$, for all frogs i. In this version of the model, there are no within-host feedback mechanisms that can regulate the build-up of sporangia on an individual host frog. The deterministic version of the Baseline Model is a set of linear ordinary differential equations, so the only two outcomes possible are exponential growth of the fungal load on each frog (see Fig. S1a)

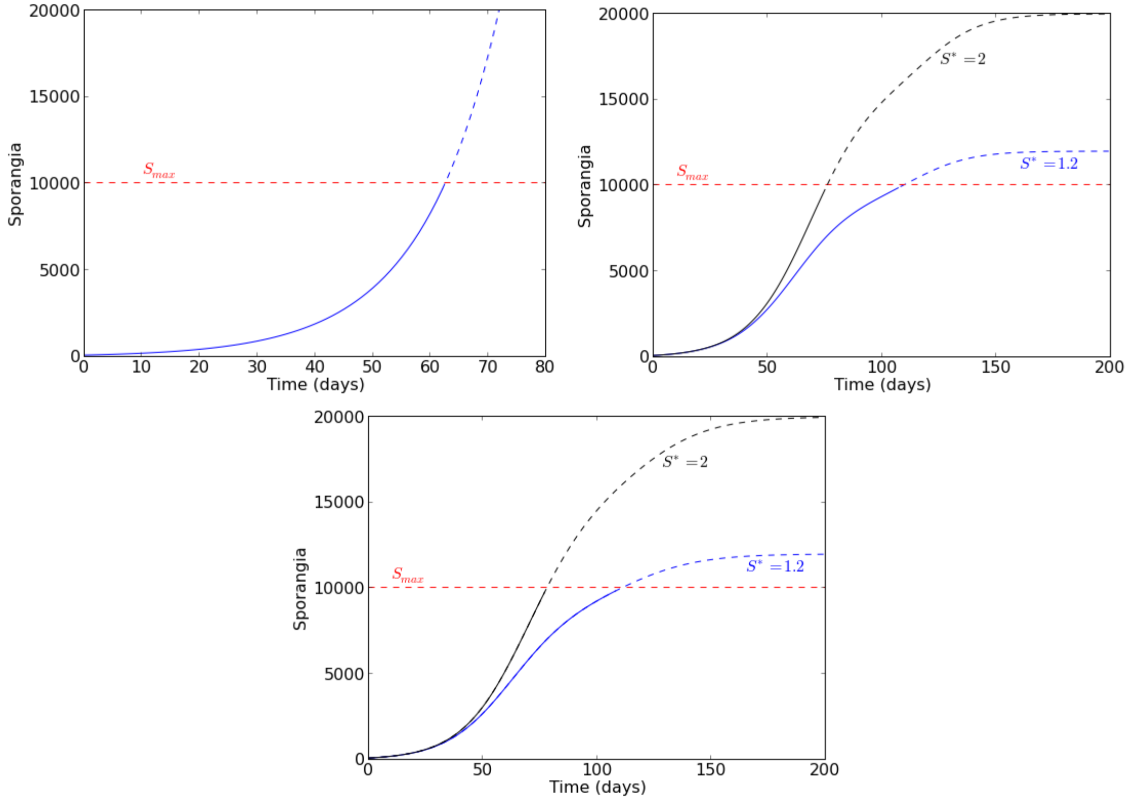

Figure S1: Example trajectories of the number of sporangia on a frog through time for the deterministic model. (A) Baseline model, (B) Sigma model, (C) Nu model. Solid lines indicate actual trajectories. The model assumes that the frog dies due to chytridiomycosis when S_i exceeds S_{max}. Dashed lines indicate the continuation of the trajectories if the frog had not died.

Table S1: Model parameters and ranges of their values. All parameters were sampled on a linear scale except for γ, which was sampled on a logarithmic scale. Z represents Zoospores, S represents Sporangia.

**Parameter**	**Range**	**Units**	**Description**
N_{frogs}	5 to 200	Frogs	Initial number of frogs
γ	10^{-6} to 10^{0}	Volume/Frog/Day	Zoospore encounter rate
η	5 to 20	$Z/S/$Day	Zoospore production rate
ν_0	0 to 1	S/Z	Zoospore encystment rate
f	0 to 1	dimensionless	Zoospore host reinfection fraction
σ_0	0.1 to 0.5	1/Day	Sporangia shedding rate
μ	0.01 to 1.5	1/Day	Zoospore death rate

until the lethal threshold ($S_i > S_{max}$) is reached [3], or exponential decline in the fungal load on all hosts and loss of infection. Which of these two outcomes occurs depends on λ, the dominant eigenvalue of the linear system (with exponential growth if $\lambda > 0$, and exponential decline if $\lambda < 0$). As described below, we selected parameter combinations for which frog extinction always occurs in the deterministic version of the model, in the absence of any interventions.

1.1.2 Nu Model (sporangia-dependent zoospore encystment): $\nu(S_i) = \nu_0 \exp(-\nu_1 S_i)$

In this variant of the model, the zoospore encystment success on each frog i is assumed to decrease as the fungal load on that frog increases. This allows for the possibility of space limitation for zoospore encystment on the frog skin, such that at high fungal loads the frog skin becomes increasingly disrupted, and the zoospores are less likely to find a suitable site on which to encyst. It may also represent a situation in which the frog's innate immune defenses, such as the production of antimicrobial peptides [4, 5] that kill zoospores that come in contact with the frog skin, increase as the intensity of infection increases. A negative exponential function is used: $\nu(S_i) = \nu_0 \exp(-\nu_1 S_i)$, so that $\nu(S_i)$ has an upper bound of ν_0 when $S_i = 0$, and approaches 0 when S_i is large (where parameter ν_1 controls the rate of decrease with increasing S_i). This variant of the model assumes a constant death rate of sporangia, $\sigma(S_i) = \sigma_0$.

In the absence of any intervention strategies, the Bd fungal load on each frog i will reach an asymptote of $S^* \times S_{max}$, where $S^* = \frac{1}{S_{max}} \frac{1}{\nu_1} \ln\left[\frac{\nu_0}{\sigma}\left(\frac{\gamma\eta(1-f)N_{frogs}}{\gamma N_{frogs}+\mu} + \eta f\right)\right]$, see Fig. S1b. If $S^* \leq 1$, the frog can tolerate the Bd infection, but if $S^* > 1$, the frog dies due to chytridiomycosis before the asymptote is reached. To determine the efficacy of the various intervention strategies, we chose parameter values for which all frogs would die due to chytridiomycosis in the deterministic version of the model, if no intervention is performed. To achieve this, we used the same set of parameter values as in the baseline

model, and adjusted the value of ν_1 so that $S^* = 1.2$ and 2.

1.1.3 Sigma Model (sporangia-dependent sporangia mortality): $\sigma(S_i) = \sigma_0 + \sigma_1 S_i$

In this variant of the model, the death rate of sporangia on the frog skin increases with increasing fungal load. This could represent increased sloughing of frog skin from highly infected individuals (as has been observed in a number of species [6]). A linear form for this function is assumed in which σ_0 is the minimum sporangium death rate that occurs when $S_i = 0$, and σ_1 is the slope: $\sigma(S_i) = \sigma_0 + \sigma_1 S_i$. In this variant of the model we assume that the zoospore encystment success is constant, $\nu(S_i) = \nu_0$, as in the Baseline model.

Similar to the Nu model, a sporangia-dependent death rate of sporangia leads to the Bd fungal load trajectory on each frog i reaching an asymptote at $S^* \times S_{max}$, where $S^* = \frac{1}{S_{max}} \frac{1}{\sigma_1} \left[\frac{\gamma \nu_0 \eta N_{frogs}(1-f)}{\gamma N_{frogs} + \mu} + \eta \nu_0 f - \sigma_0 \right]$, which can be either above or below the lethal threshold of S_{max}, as shown in Fig. S1c. As in the Nu model, we used the same set of parameters as in the Baseline model, but adjusted σ_1 so that $S^* = 1.2$ and 2, and in the deterministic version of the model all of the frogs die in the absence of any intervention strategies.

1.2 Metrics for evaluating simulation trajectories

To evaluate and compare the simulations of our models, we devised a set of metrics, listed in Table S2, applied to each run. One of the most important metrics is “Outcome”, which indicates whether the frogs go extinct, the fungal pathogen is removed from the system, or the frogs and Bd persist for the 1000 days of the simulation.

**Output Metric**	**Description**
daysToSmax	Number of days until the first frog dies
daysToF0	Number of days until all the frogs are dead
fracSurv	Fraction of the initial frog population that survives
daysToZ0	Number of days until the last zoospore dies
outcome	Either frog extinction, fungal extinction, or persistence

Table S2: Simulation output metrics for the deterministic model

1.3 Stochastic Formulation

Stochasticity played a very large role in the results of our one-time treatment strategies. The only strategy that had any chance of a positive effect in the deterministic version of

the model is culling, but this strategy has associated risks. Treating frogs with antifungal agents can be beneficial during a Bd outbreak only if the frog population exhibits high stochasticity in its disease outcome, and also if the population would suffer high mortality due to the disease in the absence of treatment. However, it would be difficult or impossible to quantify either of these properties of the population prior to Bd invasion. Therefore, in the absence of additional information about the population, we recommend the strategy of treating individuals with antifungal agents as the one-time conservation measure with a potential to have a beneficial effect and a low probability of negatively impacting on the population.

For the stochastic version of the model, we created a discrete stochastic Individual Based Model (IBM) that tracks the individual zoospores in the pool, each member of a population of frogs and the individual sporangia infecting each of those frogs. The state variables for the system are the number [N] of frogs alive in the pond, the number [Z] of zoospores in the pond, and the sporangia load [S_i] on each frog i. Table S3 shows the reactions for the discrete stochastic system. This model was then simulated using a variant of the Gillespie [7] stochastic simulation algorithm.

Previous Bd models [2, 8, 9] have used only ad-hoc representations of the stochasticity in the system. We chose this individual-based model formulation, rather than any of the alternative model formulations used for macroparasitic diseases that follow the number or fraction of hosts in the population currently infected with each number of parasites (e.g. [10, 11]), because the individual-based representation facilitates the comparison to empirical data for infection trajectories on individual frogs, and because in many populations there are far fewer frogs than potential infection classes.

Reaction	Propensity	State Change	Description
Z $\rightarrow$Frog$_i$	γ [Z] [N] $\nu(S_i)$	[Z]-1,[S_i]+1	Zoospore encysts on frog i
Z $\rightarrow$ Ø	γ [Z] [N] (1-$\nu(S_i)$)	[Z]-1	Zoospore fails to encyst
S_i $\rightarrow$Frog$_i$	η [S_i] f $\nu(S_i)$	[S_i]+1	Sporangia reinfects frog i
S_i $\rightarrow$ Ø	η [S_i] f (1-$\nu(S_i)$)	Ø	Sporangia fails to reinfects frog
S_i $\rightarrow$Z	η [S_i] (1-f)	[Z]+1	Sporangia produces zoospore
S_i $\rightarrow$ Ø	$\sigma(S_i)$ [S_i]	[S_i]-1	Sporangia shed from frog i
Z $\rightarrow$ Ø	μ [Z]	[Z]-1	Zoospore dies in pool
Frog$_i$ $\rightarrow$ Ø	[S_i] $\geq$ S_{max}	[# frogs]-1, S_i=0	Frog i dies

Table S3: Reactions for the discrete stochastic model.

Similar to the deterministic model, we developed metrics to evaluate the simulation trajectories. However, for the stochastic model there is the possibility that the same parameter point, will produce different outcomes for different realizations, therefore we instead simulate an ensemble (N=30) of stochastic trajectories and evaluated the output metrics for the entire ensemble. Compared to the deterministic simulations, this changes

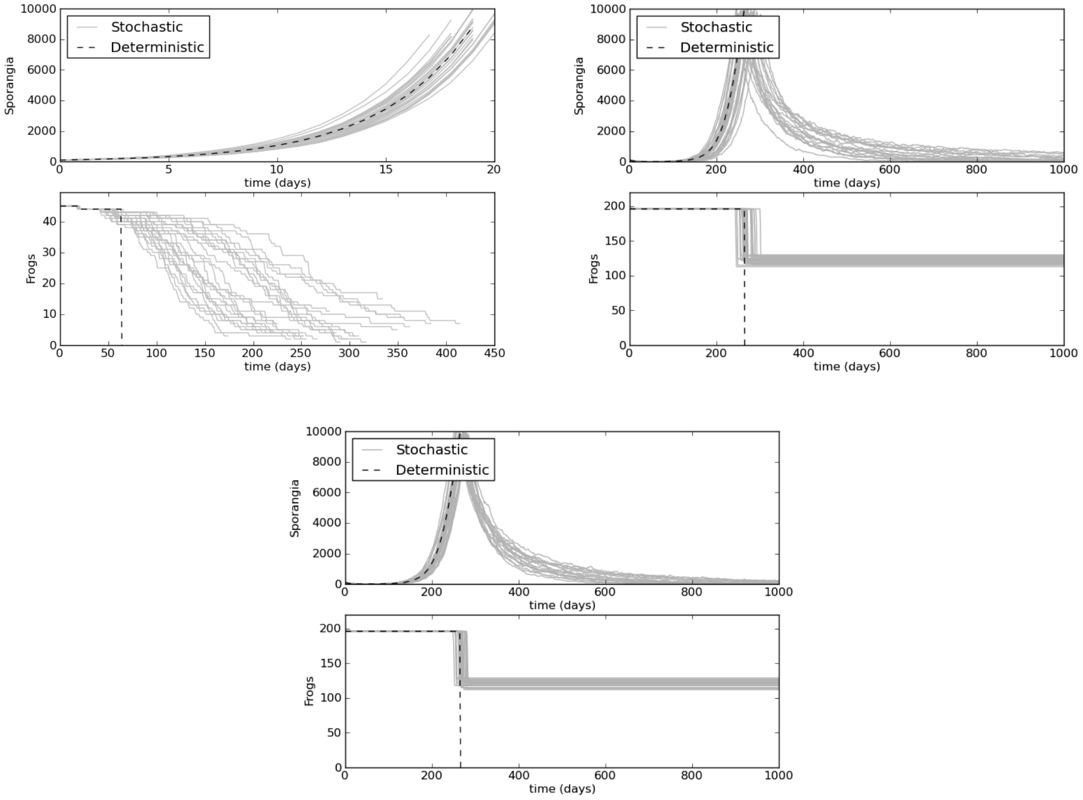

Figure S2: Stochastic simulation of the three models (solid gray lines) alongside their deterministic counterparts (dashed black line). **(A)** Baseline model showing trajectories of the sporangia on a single frog (top panel) and living frogs over time (bottom panel). **(B)** Sigma model. **(C)** Nu model.

the output metric from a single value, to a mean and standard deviation (stddev) of a distribution of values for the ensemble. For example, we track the mean days until the first frog dies (meanDaysToSmax) and the standard deviation of the days until the first frog dies (stddevDaysToSmax). For the "Outcome" output metric, we track the fraction of the total trajectories that end in each outcome: fracFrogExtinct is the fraction of trajectories for which the frogs were driven extinct (within 1000 days), FracFungusExtinct is the fraction of the trajectories where the fungus fades out, and fracPersistence is the fraction of trajectories for which the frogs and fungus are both present at the end of the 1000 day simulation. This is shown in Table S4.

**Output Metric**	**Description**
meanDaysToSmax / stddevDaysToSmax	Mean and standard deviation of the number of days until the first frog dies
meanDaysToF0 / stddevDaysToF0	Mean and standard deviation of the number of days until all the frogs are dead
meanFracSurv / stddevFracSurv	Mean and standard deviation of the fraction of the initial frog population that survives
meanDaysToZ0 / stddevDaysToZ0	Mean and standard deviation of the number of days until the last zoospore dies
fracFrogExtinct	Fraction of trajectories where the outcome is frog extinction
fracFungusExtinct	Fraction of trajectories where the outcome is fungal clearance
fracPersistence	Fraction of trajectories where the outcome is persistence

Table S4: Simulation output metrics for the stochastic model formulation

In our formulation, we take the volume of the system to be constant and vary the number of frogs. Classically, when comparing the deterministic and stochastic systems, the volume determines the relative magnitude of stochasticity. In this formulation the stochasticity goes to zero as the volume goes to infinity, however as we increase the volume we would also increase number of frogs to keep the frog density constant. We set the volume to unity and vary the number of frogs to alter the level of stochasticity.

1.4 Parameterization

We sampled this seven dimensional parameter space using a Monte-Carlo rejection method to be as inclusive as possible with all possible dynamics of the model. Table 1 (in the main text) and Table S1 shows each parameter and its corresponding range. The populations sizes come from field surveys in the Sierra Nevada[12].

The procedure we used was to randomly select a value for each of the parameters shown in main text Table 1. We then calculated the linearized Bd growth rate, which is the maximum real eigenvalue $\lambda = \max\left[\Re\left[eig\left(\mathbf{M}\right)\right]\right]$ of the system of linear equations $\mathbf{M}$ defined by equations (1-2). If the value of λ fell outside the range 0.035 to 0.35 (experimentally determined), the parameter point was rejected. We accepted 100,000 of these parameter points.

Because our model is over parameterized, and because we needed to ensure that the dynamic behavior of the system was evenly sampled, we converted the system (1-2) to non-dimensional form [13] which yielded the following set of independent parameters

$$c1 = \eta\nu f\sigma^{-1} \tag{3}$$
$$c2 = \gamma\eta(1-f)\nu\, N_{\text{frogs}}\, \sigma^{-2} \tag{4}$$
$$c3 = \gamma\, N_{\text{frogs}}\, \sigma^{-1} \tag{5}$$
$$c4 = \mu\sigma^{-1}, \tag{6}$$

and the non-dimensionalized system of equations

$$\frac{d\hat{S}_i}{d\hat{t}} = \hat{Z} + c1\hat{S}_i - \hat{S}_i \qquad \text{for} \qquad \hat{S}_i \leq \hat{S}_{max} \tag{7}$$
$$\text{frog } i \text{ dies} \qquad \text{for} \qquad \hat{S}_i > \hat{S}_{max} \tag{8}$$
$$\frac{d\hat{Z}}{d\hat{t}} = c2\sum_i \hat{S}_i - c3\hat{Z} - c4\hat{Z} \qquad i \in [1, N_{frogs}]. \tag{9}$$

The physical interpretations of these parameters are given by: $c1$ is the self reinfection rate (rate at which the disease progresses within an individual), $c2$ is the infectiousness of the frogs, and $c3$ and $c4$ are the zoospore loss rates due to interacting with the frogs and the background loss rate respectively.

We then used these formulas to find the maximum and minimum possible values for each of the four non-dimensional parameters. Using these, we find the range for each non-dimensional parameter $cN_{range} = cN_{max} - cN_{min}\ \forall N \in (1,2,3,4)$. We define the distance metric between two parameter points i and j as

$$d_{ij} = \sqrt{\left[\frac{c1_i - c1_j}{c1_{range}}\right]^2 + \left[\frac{\log(c2_i) - \log(c2_j)}{\log(c2_{range})}\right]^2 + \left[\frac{\log(c3_i) - \log(c3_j)}{\log(c3_{range})}\right]^2 + \left[\frac{c4_i - c4_j}{c4_{range}}\right]^2}. \tag{10}$$

Using this metric, we found the distance from the new parameter point i to the set of all previously accepted parameter points J. If $\min_{j\in J}\left[d_{ij}\right] > \epsilon$, then we accepted i into J. We repeated this procedure until J had a sufficient number of parameter points to cover our parameter space. We used $\epsilon = 0.09$, which gave us 2132 unique points. Fig. S3 and Fig. S4 illustrates the parameter space sampling.

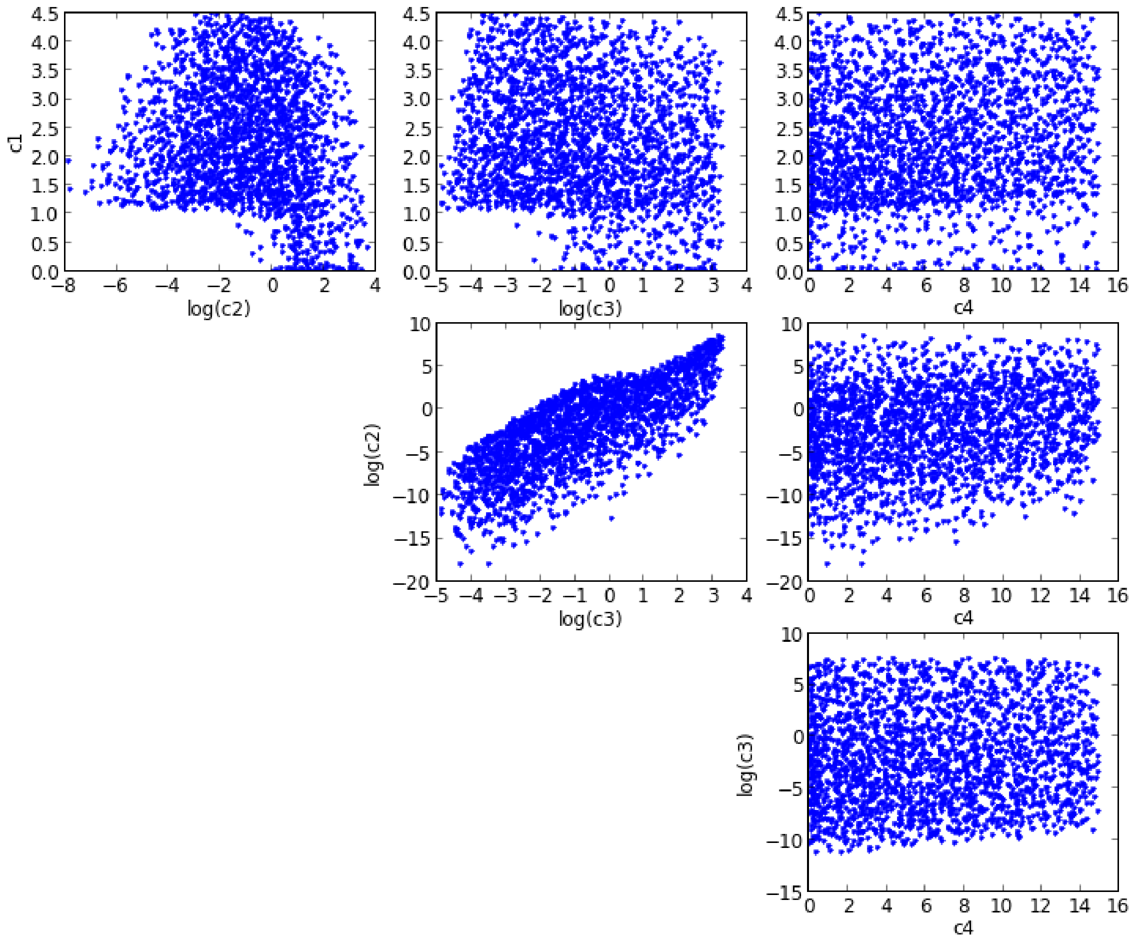

Figure S3: Sampling of the non-dimensionalized parameter space.

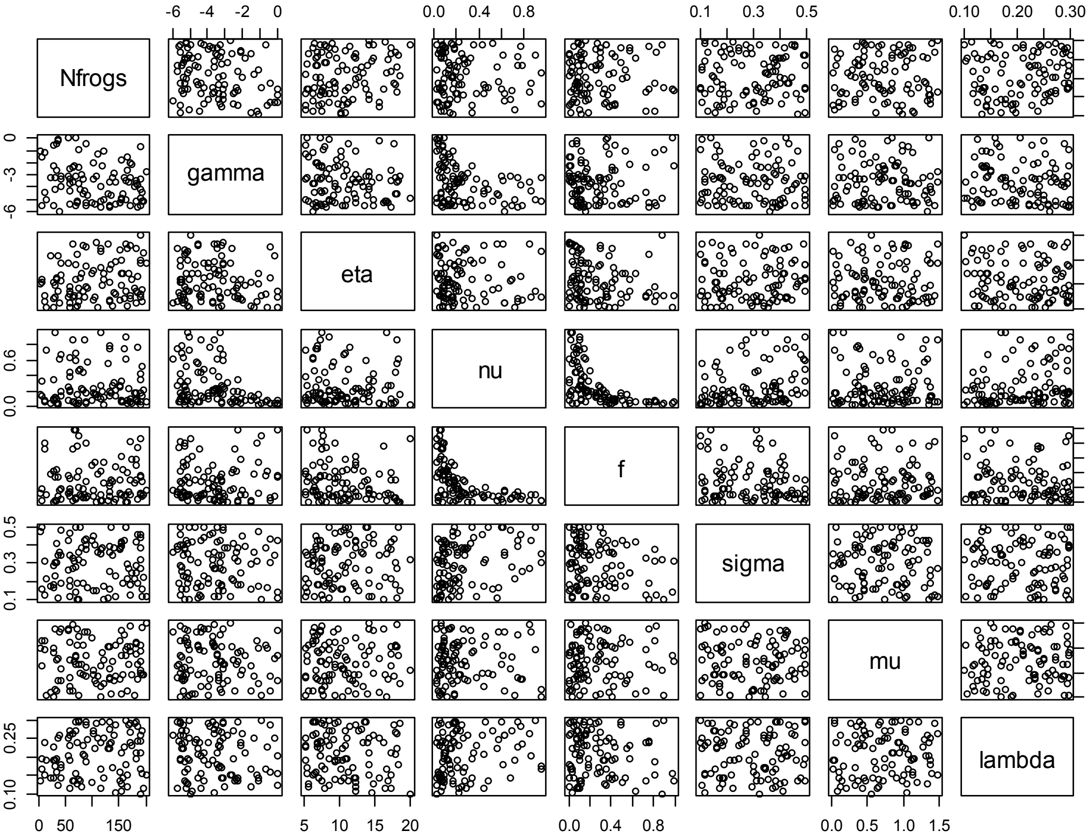

Figure S4: Sampling of the original parameter space. Random subsample of 2132 points shown.

For the Nu and Sigma models, the number of sporangia on each frog approaches an equilibrium value, $S^* \times S_{max}$, monotonically. If $S^* < 1$ for a given set of parameters and initial number of frogs, then there will be no mortality (in the deterministic formulation) and the infected frogs will survive indefinitely. In this case, no treatment strategies are necessary. The only situation in which any of the treatments have a positive effect is if the equilibrium sporangia load is greater than S_{max}, ($S^* > 1$). We chose $S^* = 1.2$ and $S^* = 2$. We can then find the parameter ν_1,

$$\nu_1 = \frac{1}{S^* S_{max}} \left(\frac{\nu_0}{\sigma} \left(\frac{\gamma\eta(1-f)\mathrm{N}_{frogs}}{(\gamma \mathrm{N}_{frogs} + \mu)} \right) \right) \tag{11}$$

and the parameter σ_1,

$$\sigma_1 = \frac{1}{S^* S_{max}} \left(\frac{\gamma\nu\eta(1-f)\mathrm{N}_{frogs}}{(\gamma \mathrm{N}_{frogs} + \mu)} + \eta\nu f - \sigma_0 \right). \tag{12}$$

The Nu and Sigma models have two different values of S^*, for a total of 4,264 parameter points in each.

The total number of parameter points for all models without conservation is 10,660. Because each of these parameter points was run with each of the three conservation methods, each having two parameters (day and efficacy of intervention) with three values, this results in a total number of points within our parameter space of 298,480.

We base our conclusions on the completeness of our computational study for the particular scenario we investigated (i.e. invasion of Bd into a naïve single host system such as *Rana muscosa* in a Sierra Nevada pond). In our modeling effort we attempted to include all possible dynamics and reduce the number of assumptions we made on our system. Additionally, we took measures to ensure that we had uniform sampling of the disease dynamics, to prevent biasing of our results by over-sampling any particular region of the space. The dynamic space which we computed was large and the associated computational effort required more than 60 CPU-years.

1.5 Stochastic Convergence

To determine whether 30 simulations was sufficient to accurately characterize the empirically sampled probability distributions for the model output metrics, we performed a convergence study and examined how the values of three different output metrics changed as we varied the ensemble size. Figure S5 shows the results from our stochastic convergence study. The output metrics do not show signifiant changes as the ensemble size is increased.

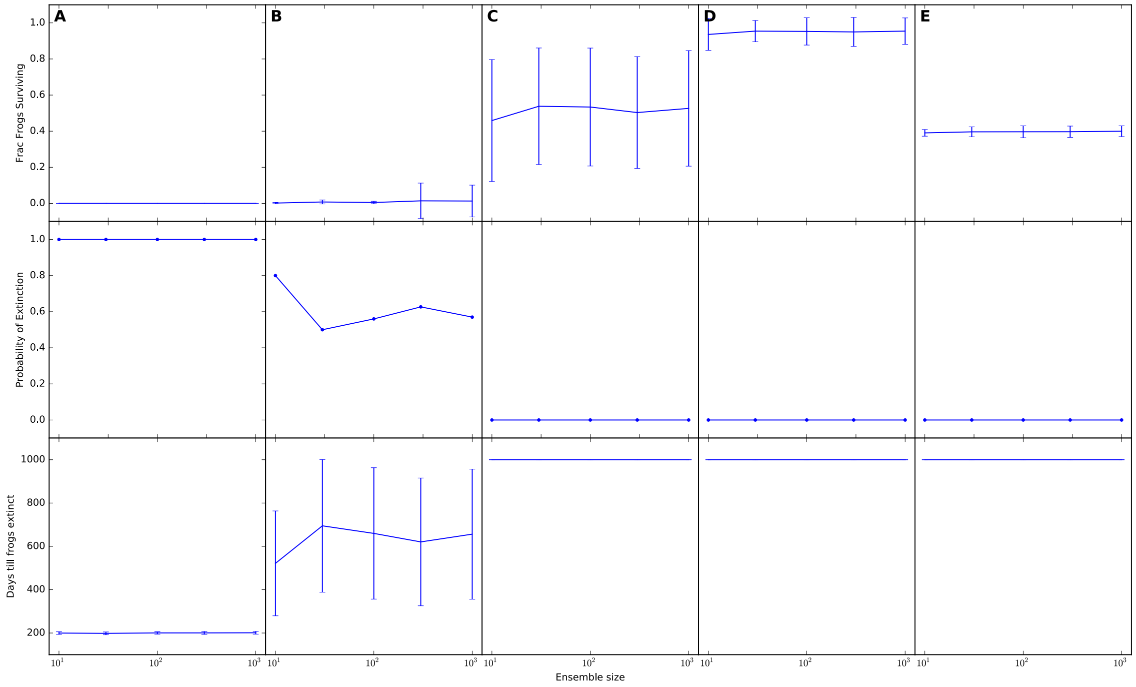

Figure S5: Stochastic convergence study for the representative points from the parameter space groups.

2 Statistical Analysis

2.1 Correlation

We calculated correlation coefficients between the input parameters and the output metrics for the baseline model without interventions. In most cases we found weak correlations ($|r| < 0.2$). Table S5 shows the correlations that were signifiant. We also looked at which conservation parameters correlated with the output metric (see Table S6).

Parameter	Metric	Value
Nfrogs	meanFracSurviving	-0.234
	meanDaysToZ0	0.258
	fracFrogsExtinct	0.208
	fracFungusExtinct	-0.241
gamma	meanFracSurviving	-0.206
	meanDaysToZ0	0.226
	meanDaysToF0	-0.221
	fracFrogsExtinct	0.204
	fracFungusExtinct	-0.227
nu	meanDaysToZ0	0.201
f	meanFracSurviving	0.318
	meanDaysToZ0	-0.328
	meanDaysToF0	0.221
	fracFrogsExtinct	-0.219
	fracFungusExtinct	0.309

Table S5: Significant ($|r| > 0.2$) correlations between input parameters and output metrics for the baseline model without interventions. All other comparisons were less significant ($|r| < 0.2$).

2.2 MIC Analysis

Table S7 shows the value of the Maximal Information Coefficient (MIC)[14] for the impact of the non-dimensional parameters on the output metrics for the baseline model without intervention. MIC is a non-parametric approach for evaluating the significance of the (potentially non-linear) relationship between two variables. The five most signifiant MIC values are shown in bold. Fig. S6 shows the plots of the parameter points versus the output metrics for these values. From these results, we see that the parameter $c1$ is most predictive of the time until the first frog dies (meanDaysToSmax), $c2$ is most predictive of the survival outcome (fracFrogsExtinct and fracFungusExtinct), disease impact (meanFracSurviving),

	meanDaysToSmax	stdDaysToSmax	meanFracSurviving	stdFracSurviving	fracFrogsExtinct
clean_time	0.169	0.174	0.057	0.043	0.009
clean_frac	0.395	0.391	0.093	0.143	-0.007
cull_time	-0.172	-0.163	-0.064	0.009	-0.027
cull_frac	-0.203	-0.175	-0.105	0.021	-0.060

Table S6: Correlations between conservation parameters and output metrics for the baseline model. Cleaning extends the time and broadens the distribution of when the first frog dies (mean/std DaysToSmax). Culling reduces the time and variance of when the first frog dies. None of the conservation methods correlate strongly with the fraction of surviving frogs or the probability that the frogs go extinct.

and disease clearance rate (meanDaysToZ0). Interestingly, $c3$ and $c4$ are not powerful predictors for any metrics.

2.3 Global Sensitivity Analysis

We conducted a variance-based global sensitivity analysis (GSA), High-Dimensional Model Representation (HDMR) [15, 16, 17, 18], to examine the effects of the four non-dimensional parameters on the output metrics of interest. The goal of our global sensitivity analysis was to find the partial variance of an output metric due to the effect of a particular parameter. This is done by fixing the value of that parameter and quantifying the resulting reduction in the total variance of the output measure. We then ranked the sensitivity indices to determine the relative importance of that parameter on the output metric.

To apply this approach to our Monte-Carlo simulation results, we sliced the individual parameter ranges into bins. We took the mean value (for a particular output metric) for all the data points within each bin and then calculated the variance of all the means across the all the bins. This method produces the partial variance of the output data with respect to that parameter. The sensitivity indices were found by dividing the partial variances by the total variance. This procedure calculates the first-order sensitivities. Higher-order sensitivities are also available, and all of the sensitivities should sum to unity. We report the sum of the first-order sensitivities to show their total contribution to the global sensitivity.

One challenge for this method is that as the number of bins increases, the number of data points per bin decreases, and in some cases drops to zero. The result is that the mean

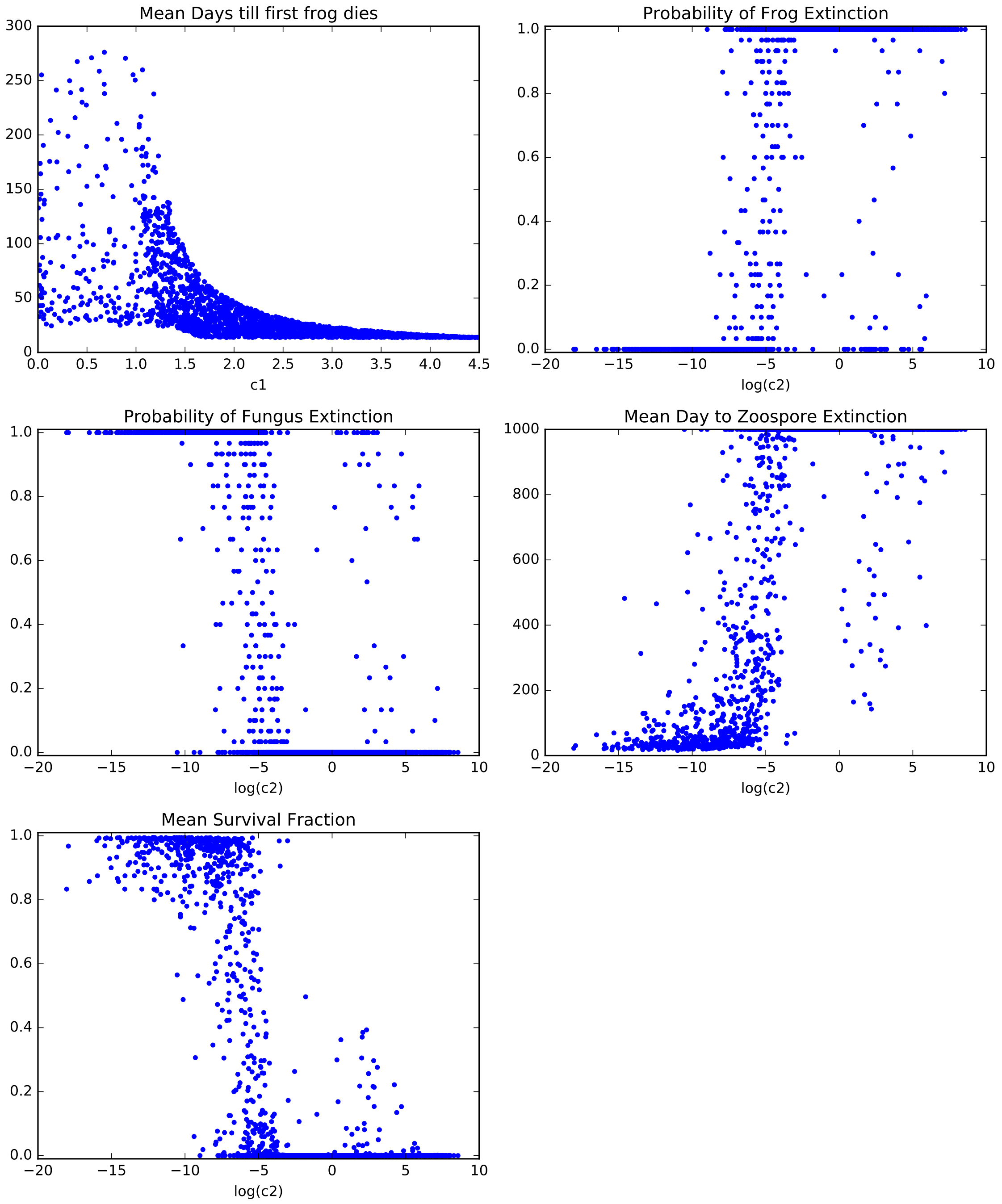

Figure S6: Plots of the parameter points versus the output metrics for the five most significant values of the MIC analysis on the baseline model without interventions.

	c1	c2	c3	c4
fracFrogsExtinct	0.10	**0.68**	0.33	0.10
fracFungusExtinct	0.11	**0.68**	0.33	0.10
fracPersistence	0.10	0.11	0.09	0.10
meanDaysToF0	0.13	0.59	0.30	0.16
meanDaysToSmax	**0.65**	0.16	0.12	0.12
meanDaysToZ0	0.11	**0.70**	0.33	0.10
meanFracSurviving	0.11	**0.68**	0.33	0.10
stdDaysToF0	0.11	0.60	0.29	0.19
stdDaysToSmax	0.51	0.10	0.10	0.10
stdDaysToZ0	0.11	0.68	0.33	0.10
stdFracSurviving	0.11	0.56	0.26	0.10

Table S7: Value of the Maximal Information Coefficient (MIC) for each of the non-dimensional parameters, compared to the output metric for the baseline model.

value of a bin could be widely different compared to the bins close to it. Furthermore, if the bin is empty, then the partial variance is impossible to compute. We sought to ameliorate these issues by a two-pronged strategy of interpolation and thresholding of the data. To interpolate over bins that contain no data we employed the LOWESS (Locally Weighted Scatterplot Smoothing) regression method [19, 20]. This is a nonlinear least-squares regression function that takes locally weighted subsets of the data to build a quadratic polynomial model, giving more weight to points near the point whose response is being estimated and less weight to points further away. We then removed data bins from the set that contained less than a threshold number of points, as bins affect the variance disproportionately. The combined effect of these two techniques was to allow a large fraction of the data to be analyzed without the irregularity of the output in sparse regions of parameter space.

Figure S7 illustrates the sensitivity of the mean fraction of frogs surviving in the Baseline model without conservation strategies, to the four non-dimensional parameters. This output metric is highly sensitive to $log(c2)$ and $log(c3)$, and relatively insensitive to $c1$ and $c4$. Figure S8 shows an example of the sensitivity in response to a conservation strategy (culling 50% of the population at 28 days). In this case, the y-axis is the difference between the mean fraction of the frogs surviving with and without the conservation strategy (positive value means more frogs survive, and negative value means fewer frogs survive). This metric is highly sensitivity to $log(c2)$ and $log(c3)$, with culling having a strong negative effect for low values of these parameters and a positive effect for high values of these parameters. Tables S8 shows the sensitivities of the difference in the mean fraction of frogs surviving with and without intervention strategies in the stochastic Baseline model for the cleaning and culling conservation strategies. Data for cleaning the environment and for the Nu and Sigma variants of the model are not shown in the sensitivity analysis results,

**Day**	**Fraction**	$\mathbf{S_1}$	$\mathbf{S_2}$	$\mathbf{S_3}$	$\mathbf{S_4}$	$\mathbf{S_{total}}$
**clean**						
14	25%	0.317	0.352	0.172	0.042	0.883
14	50%	0.214	0.501	0.139	0.038	0.892
14	75%	0.148	**0.652**	0.121	0.039	0.96
28	25%	0.28	0.382	0.161	0.032	0.855
28	50%	0.254	0.431	0.133	0.031	0.849
28	75%	0.231	0.592	0.124	0.021	0.968
56	25%	0.265	0.411	0.14	0.031	0.847
56	50%	0.231	0.507	0.122	0.041	0.901
56	75%	0.221	0.552	0.111	0.041	0.925
**cull**						
14	25%	0.038	**0.697**	0.2	0.016	0.951
14	50%	0.051	**0.687**	0.188	0.019	0.945
14	75%	0.042	**0.771**	0.151	0.022	0.986
28	25%	0.046	**0.702**	0.18	0.012	0.94
28	50%	0.051	**0.727**	0.158	0.019	0.955
28	75%	0.048	**0.759**	0.157	0.021	0.985
56	25%	0.039	**0.712**	0.166	0.018	0.935
56	50%	0.042	**0.731**	0.156	0.012	0.941
56	75%	0.044	**0.745**	0.149	0.019	0.957

Table S8: First-order sensitivity indices for non-dimensional parameters for the difference in the mean fraction of frogs surviving with and without intervention strategies in the stochastic baseline model. S_1, S_2, S_3, and S_4 are the sensitivities for the parameters $c1$, $log(c2)$, $log(c3)$, and $c4$ respectively. S_{total} is the sum of S_1, S_2, S_3, and S_4. Significant sensitivities (> 0.6) are shown in bold.

for brevity.

Our analysis of all variants of the model shows that almost all of the output metrics are relatively insensitive to the parameter $c4$. Parameter $c1$ has more influence in the deterministic models, whereas $log(c2)$ and $log(c3)$ have a greater effect on the sensitivities for the stochastic models. Some output metrics do not have an easy interpretation for global sensitivity analysis because the metric is invariant with respect to the input parameters. For example, the times to the fade out of the fungus (daysToZ0) and the fraction of frogs surviving (fracSurv) always have the same result in the deterministic Baseline model, which is that the frogs are driven to extinction.

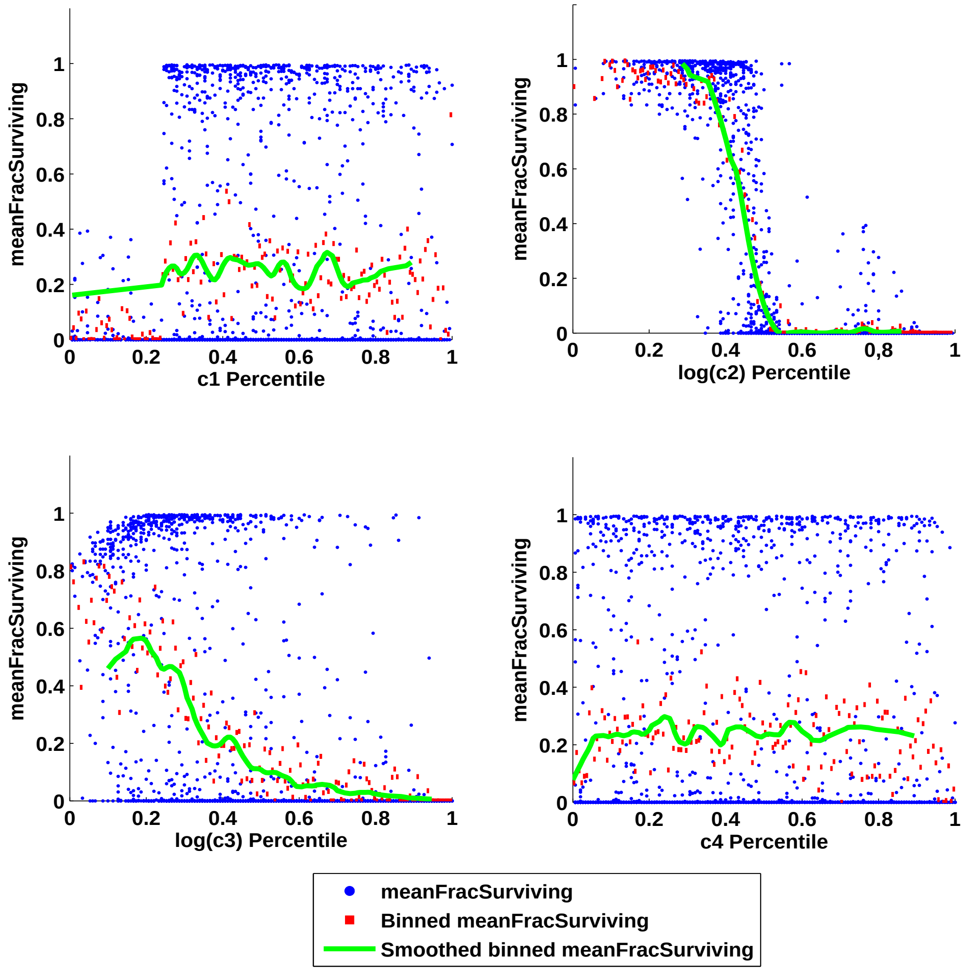

Figure S7: First-order sensitivity indices for the fraction of frogs surviving without conservation in the stochastic baseline model. Blue points are the simulation data, red points are the mean of each bin, and the green line is the LOWESS interpolation of the mean values.

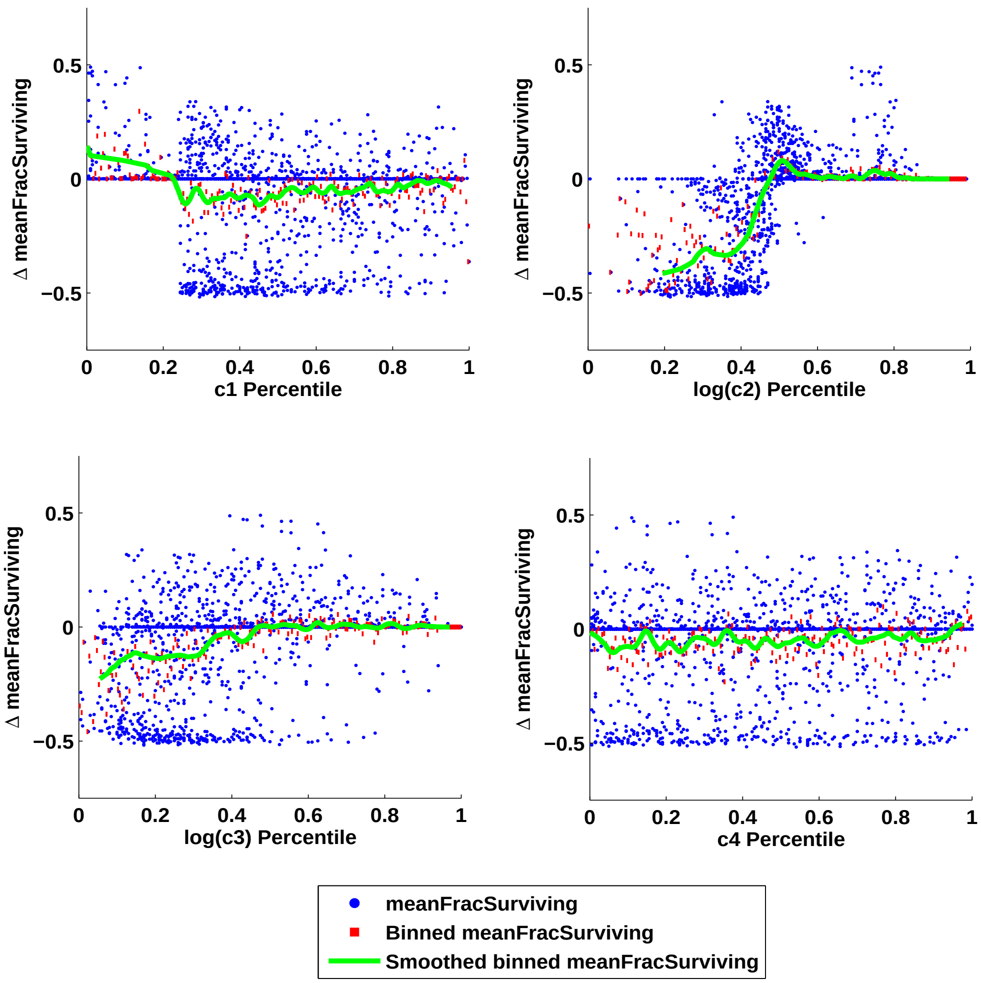

Figure S8: First-order sensitivity indices for the difference in the fraction of frogs surviving with and without culling 50 percent of the frogs at 28 days in the stochastic baseline model. Blue points are the simulation data, red points are the mean of each bin, and the green line is the LOWESS interpolation of the mean values.

3 Subdivision of the dynamical space into groups

We subdivided the parameter points into 5 groups by examining the parameter space in terms of the mean fraction of the frog surviving (meanFracSurviving) versus the standard deviation of frogs that survive (stdFracSurviving). Fig. S9 is an expanded view of main text Fig. 4, with each group shown separately. The parameters for the five representative points shown in main text Fig. 4 are shown in Table S9.

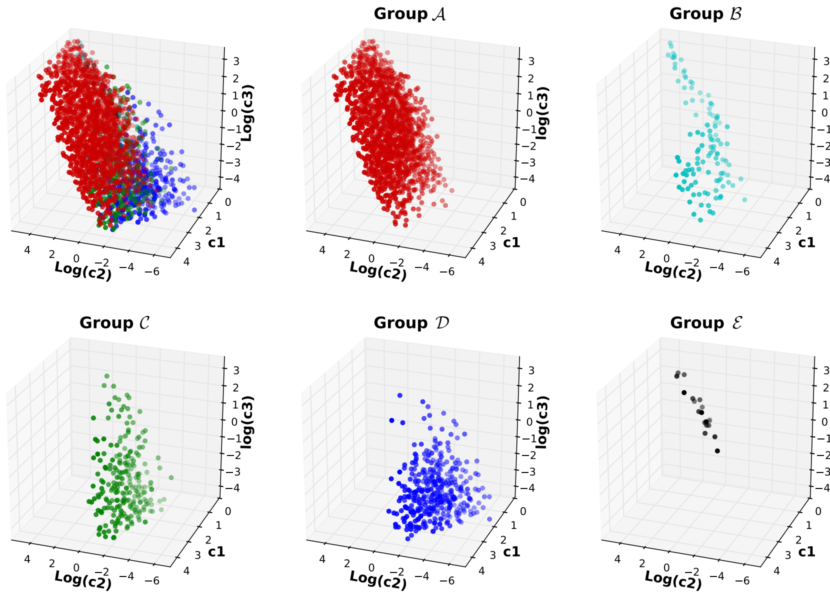

Figure S9: Distribution of Groups $\mathcal{A}, \mathcal{B}, \mathcal{C}, \mathcal{D}$, and $\mathcal{E}$ in nondimensionalized c1-log($c2$)-log($c3$) parameter space.

3.1 Sensitivity of Conservation Measures

Figure S10 shows the results from sensitivity analysis of the conservation parameters.

Group	ID	N_{frogs}	γ	η	ν	f	σ	μ
A	120	194	8.31e-5	5.01	0.771	0.069	0.254	1.42
B	1094	111	7.83e-4	11.38	0.026	0.992	0.192	1.23
C	182	85	3.19e-6	11.61	0.078	0.560	0.201	1.25
D	982	142	2.45e-6	7.299	0.105	0.711	0.313	1.49
E	192	127	4.51e-3	5.67	0.111	0.054	0.181	1.21

Table S9: Parameters for the five representative points from the parameter space groups.

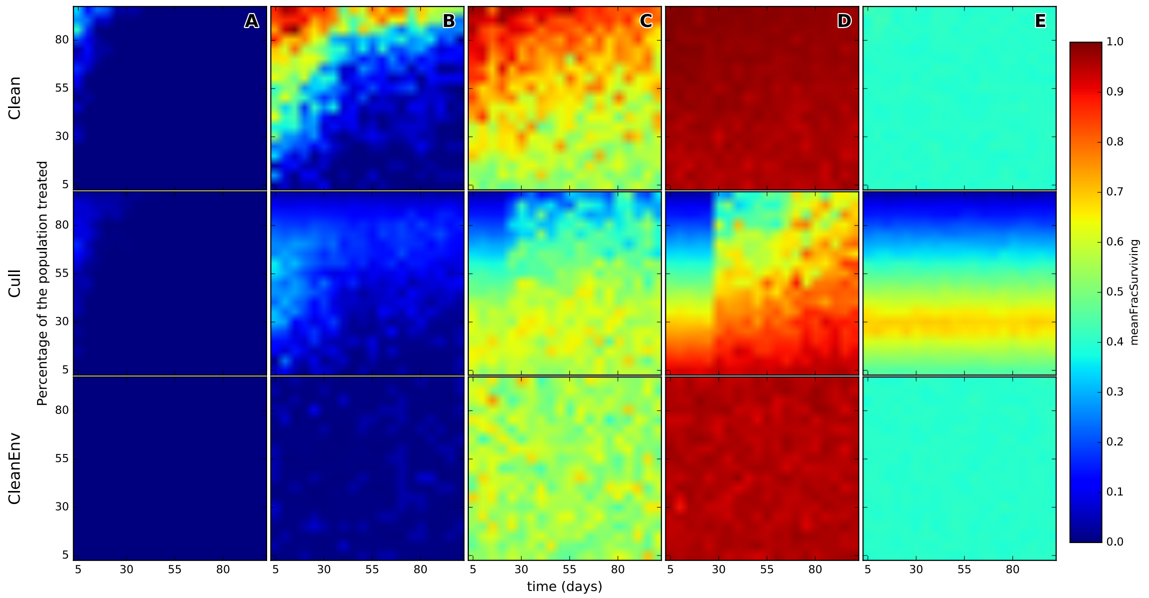

Figure S10: Sensitivity of Conservation Measures examined on the five representative points for the varying effect of changing the conservation parameters of time of application and effectiveness of treatment for the three conservation methods: cleaning, culling and cleaning the environment. Times of application are 5,10, $\cdots$ 100 days. Effectiveness are 5%, 10%, $\cdots$ 95%. The color represents the mean fraction of frogs that survive, with red corresponding to 100%, blue to 0%.

4 Additional Conservation Results

4.1 Conservation by Permanent Alteration of the Environment

Whereas the three mitigation strategies presented in the main text are pulse treatments applied to the system, in this category we lump mitigation strategies aimed at fundamentally altering the host pathogen dynamics through changing the parameters of the interaction. In this category we include biological control or probiotics, which may alter the survival of Bd in the environment or increase the long-term resistance or tolerance of hosts to infection. Biological control approaches based on the release of predators, such as *Daphnia* [21, 22, 23, 24], that can consume the aquatic zoospore stage, have been proposed as a means of reducing the survival of Bd zoospores in the environment. Field trials of this type of biological control have not been attempted, and given the potential for complex dynamics of *Daphnia* populations [25], it is unclear whether such predators can be maintained as long-term biological control agents. Schmeller et al. [22], however, showed that across field sites, the densities of naturally-occurring predatory microorganisms were negatively correlated with the impact of Bd on one amphibian species. The potential for use of probiotic bacteria to protect amphibians from chytridiomycosis has received a great deal of recent attention [26, 27, 28, 29, 30]. In a number of laboratory experiments, treatment of amphibians with specific bacteria species has reduced the susceptibility of the amphibians to subsequent exposure to Bd, while other such attempts have been unsuccessful.

We implemented permanent alteration conservation methods of intervention by permanently changing one or more parameters of the model. An example would be the introduction of a predator (such as *Daphnia*) that eats the zoospores in the pond [23, 24, 22, 21]. This would effectively increase the value of μ, the average zoospore death rate. Another would be to alter the flow rate of streams flowing into and out of the pond, which could change both μ and f. A third example would be the introduction of a probiotic bacteria on the skin of the frogs that decreases the susceptibility of the frogs to zoospore encystment, effectively decreasing ν [31, 32, 33].

To examine whether these strategies could affect the survival outcome of a frog population during an outbreak, we investigated, for each parameter combination, how much each parameter in the baseline model would need to be altered in order to drive the growth rate λ of the disease from a positive to a negative value. If the growth rate becomes negative, the disease will fade out, resulting in a positive outcome for the frog population. For N_{frogs}, γ, η, ν and f, we explored the outcome as a function of the fraction by which the parameter was reduced from its original value. For σ and μ, we explored the outcome as a function of the fraction by which the *inverse* of the parameter is reduced, as these parameters represent the inverse of average lifespan of sporangia and zoospores, respectively.

Fig. S11 shows the fraction of the parameter space for which the growth rate becomes negative as a function of the amount by which each parameter is reduced. It shows that for γ and μ^{-1} (red line), there is almost no reduction amount that will lead to a positive

outcome. For N_{frogs} (black line), only for very large reductions do we see a positive outcome. In contrast, λ is highly sensitive to reductions in η, ν, and σ^{-1} (blue line). Reducing these parameters by 70% or more leads to a positive outcome for all parameter combinations. We also see that a large reduction in the fraction f of reinfection (orange line) leads to a positive outcome in approximately 60% of the cases.

Figure S12 shows the effect of reducing the parameter values on the fraction of frogs that survive, and probability of the frogs going extinct for the stochastic model using the representative points from the parameter space groups. Note that the group $\mathcal{A}$ results are consistent with deterministic analysis.

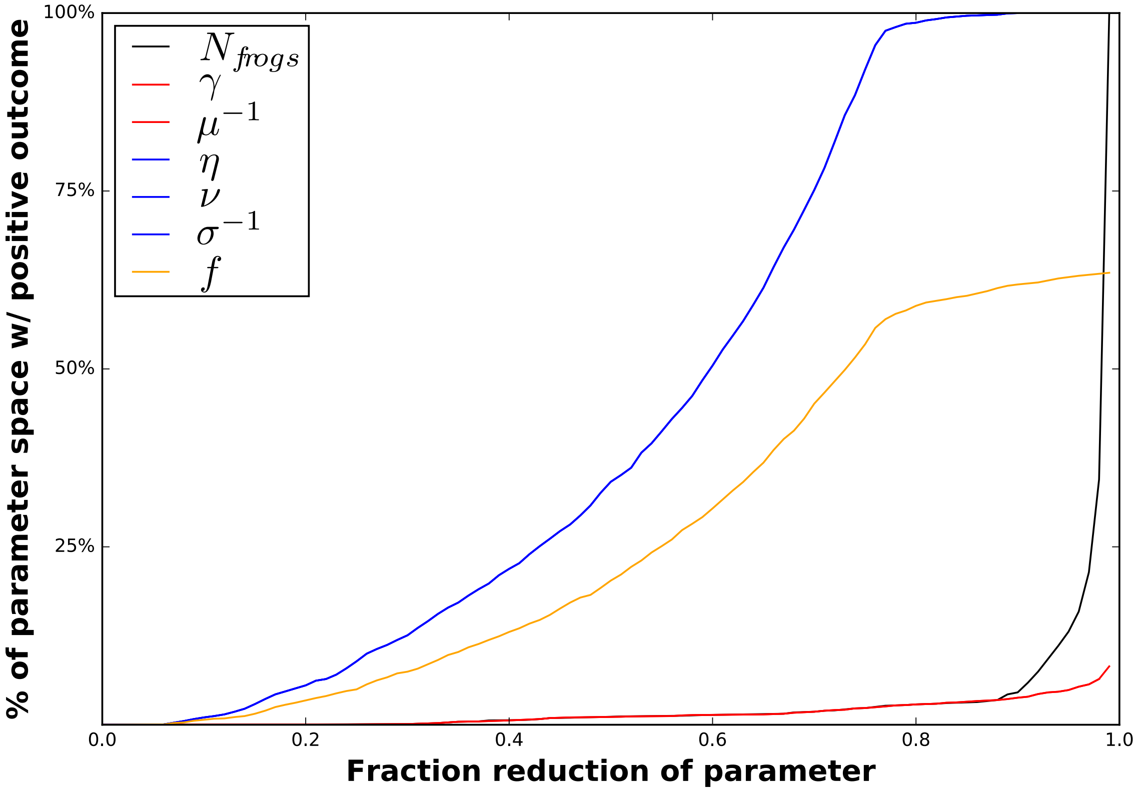

Figure S11: Sensitivity of the outcome of the host-pathogen system to conservation through permanent alteration of the parameters. Each line represents the fraction of the parameter space where the growth rate λ of the disease goes from positive to negative as a function of the amount by which each parameter is reduced. The parameters η, ν, and σ^{-1} (blue line) display the strongest sensitivity to permanent alterations of the system. These are followed by f (orange line), N_{frogs} (black line). Finally, γ and μ^{-1} (red line) display the least sensitivity.

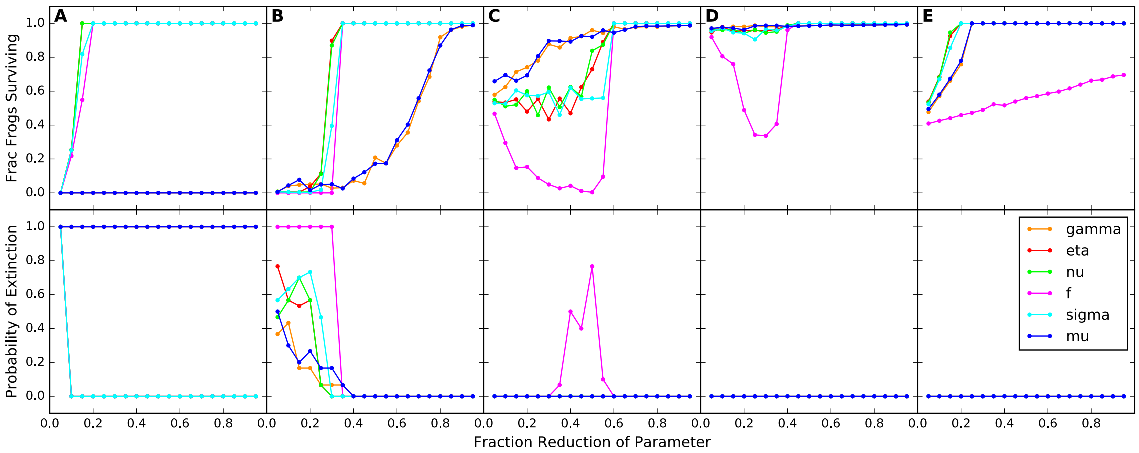

Figure S12: Conservation through permanent alteration on the stochastic model using the representative points from the parameter space groups. For gamma, eta, nu, and f, the nominal value of the parameter was multiplied by $1 - x$, where x is the fraction reduction. For sigma and mu, the nominal value was multiplied by $1/(1 - x)$.

4.2 Effectiveness of Conservation Measures

In the main text Fig. 5, we see the effect of the one-time conservation measures on the stochastic models. Fig. S13 shows the same data for the deterministic models. Fig. S14 shows the effectiveness of each of the conservation methods broken out by the day and fraction of the population treated.

We also investigated the application of multiple conservation strategies on a frog population, see figure S15. The data used to generate this figure was limited to the day of application of the treatment (after Bd introduction) and 75% effectiveness as treatment. The first three rows show a single treatment strategy, while the last three rows show the results of the application of two treatments. We observe that the application of two treatments does not significantly change the results, which closely resemble the results of the more effective single treatment strategy in the combination.

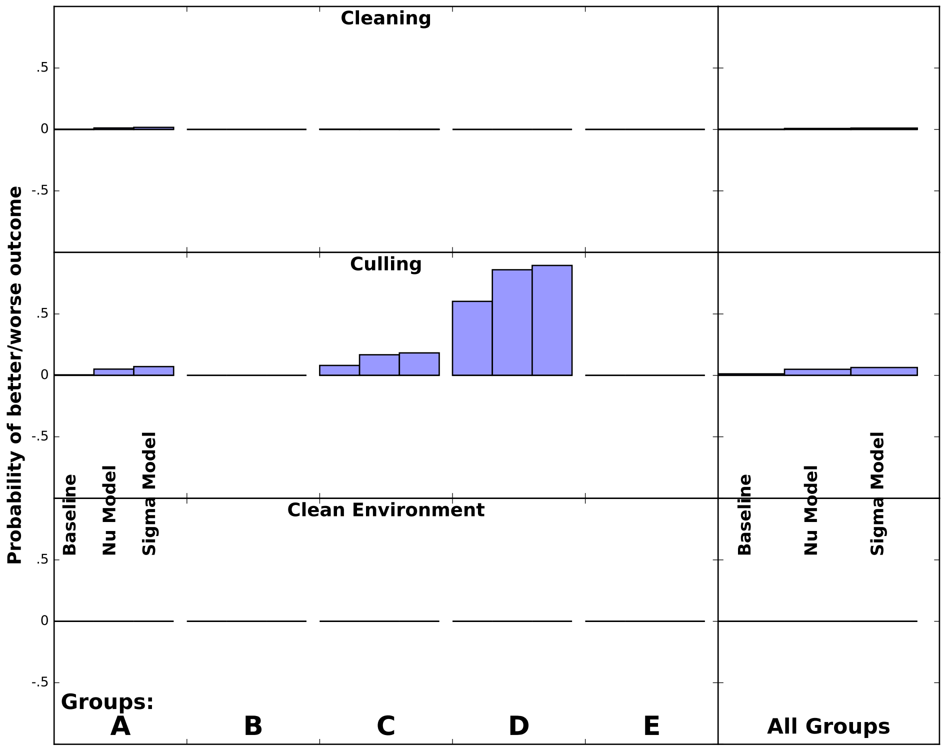

Figure S13: Effects of conservation measures on the subdivided dynamical space for the deterministic models. Blue bars show the fraction of parameter points for which application of the conservation measure improves the fraction of frogs surviving by at least 10%. Red bars show the fraction of points where the outcome is decreased by at least 10%.

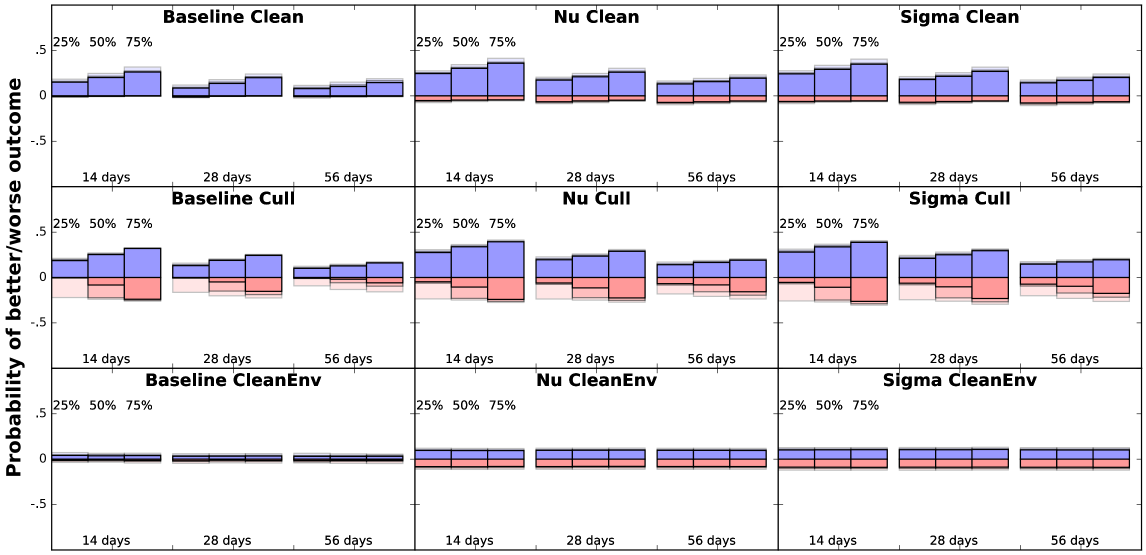

Figure S14: Effectiveness of each of the conservation methods broken out by the day and fraction of the population treated. Blue bars show the fraction of parameter points for which the application of the conservation measure improves the fraction of frogs surviving by at least 10% / 30% / 50% (light / medium / dark). Red bars show the fraction of points where the outcome is decreased by at least 10% / 30% / 50% (light / medium / dark).

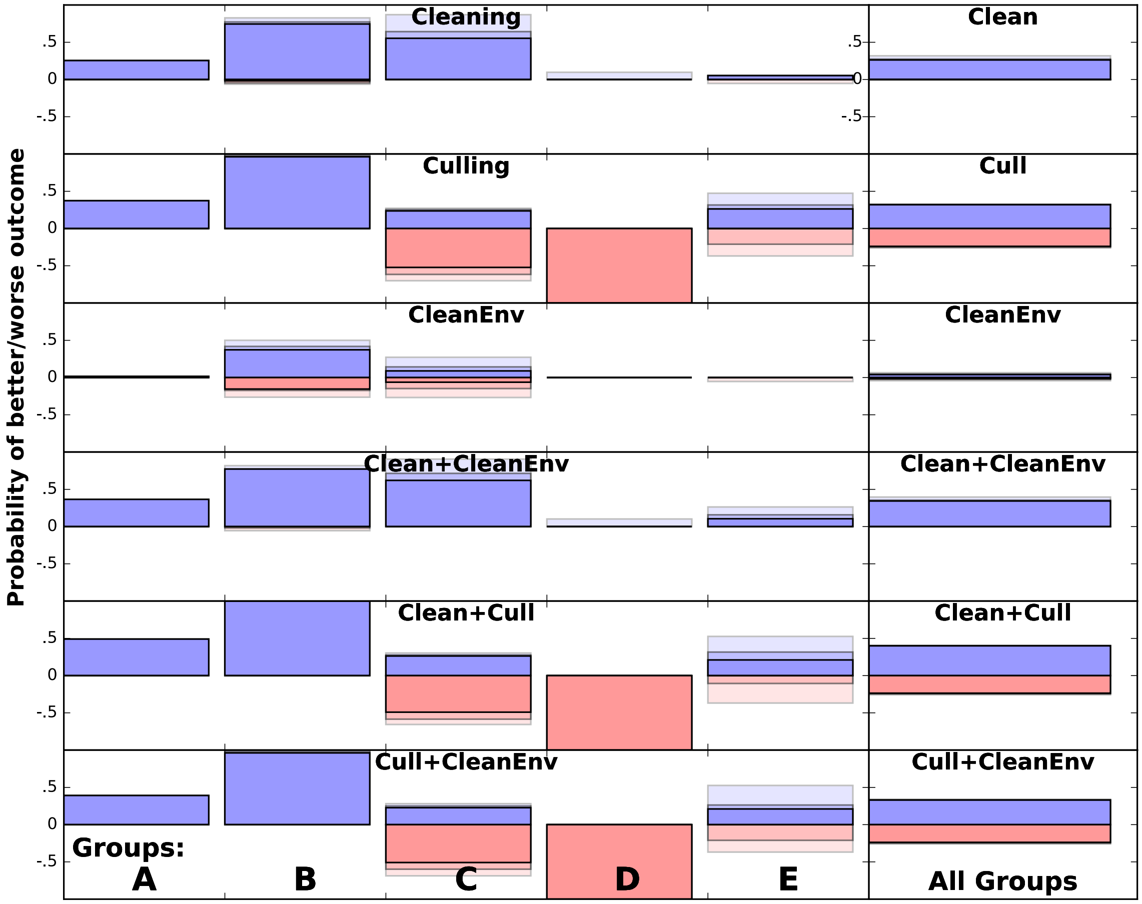

Figure S15: Effectiveness of each of the application of multiple conservation methods. This data only shows conservations applied to the baseline model, only using day 14 and 75% effectiveness for each conservation strategy. Left shows effectiveness by group (see Figure 3 in the main text), right shows effectiveness across all groups. Blue bars show the fraction of parameter points for which the application of the conservation measure improves the mean fraction of frogs surviving by at least 10% / 30% / 50% (light / medium / dark). Red bars show the fraction of points where the outcome is decreased by at least 10% / 30% / 50% (light / medium / dark). The sign (+/-) of the y-axis indicates whether the conservation strategy had a positive or negative effect.

References

[1] Gough B. GNU Scientific Library Reference Manual - Third Edition. 3rd ed. Network Theory Ltd.; 2009.

[2] Briggs CJ, Knapp RA, Vredenburg VT. Enzootic and epizootic dynamics of the chytrid fungal pathogen of amphibians. Proceedings of the National Academy of Sciences. 2010;107(21):9695–9700.

[3] Stice MJ, Briggs CJ. Immunization is ineffective at preventing infection and mortality due to the amphibian chytrid fungus Batrachochytrium dendrobatidis. Journal of Wildlife Diseases. 2010;46(1):70–77.

[4] Woodhams D, Ardipradja K, Alford R, Marantelli G, Reinert L, Rollins-Smith L. Resistance to chytridiomycosis varies among amphibian species and is correlated with skin peptide defenses. Animal Conservation. 2007;10(4):409–417.

[5] Woodhams D, Rollins-Smith L, Alford R, Simon M, Harris R. Innate immune defenses of amphibian skin: antimicrobial peptides and more. Animal Conservation. 2007;10(4):425–428.

[6] Ohmer ME, Cramp RL, White CR, Franklin CE. Skin sloughing rate increases with chytrid fungus infection load in a susceptible amphibian. Functional Ecology. 2015;29(5):674–682.

[7] Gillespie DT. A general method for numerically simulating the stochastic time evolution of coupled chemical reacting systems. Journal of Computational Physics. 1976;22:403–434.

[8] Louca S, Lampo M, Doebeli M. Assessing host extinction risk following exposure to Batrachochytrium dendrobatidis. Proceedings of the Royal Society of London B: Biological Sciences. 2014;281(1785):20132783.

[9] Doddington BJ, Bosch J, Oliver JA, Grassly NC, Garcia G, Schmidt BR, et al. Context-dependent amphibian host population response to an invading pathogen. Ecology. 2013;94(8):1795–1804.

[10] Rosà R, Pugliese A. Aggregation, stability, and oscillations in different models for host-macroparasite interactions. Theoretical Population Biology. 2002;61(3):319–334.

[11] Rosà R, Pugliese A, Villani A, Rizzoli A. Individual-based vs. deterministic models for macroparasites: host cycles and extinction. Theoretical Population Biology. 2003;63(4):295–307.

[12] Knapp RA, Matthews KR. Non-Native Fish Introductions and the Decline of the Mountain Yellow-Legged Frog from within Protected Areas. Conservation Biology. 2000;14(2):428–438.

[13] Gurney W, Nisbet RM. Ecological dynamics. Oxford University Press, Oxford; 1998.

[14] Reshef DN, Reshef YA, Finucane HK, Grossman SR, McVean G, Turnbaugh PJ, et al. Detecting novel associations in large data sets. Science. 2011;334(6062):1518–1524.

[15] Sobol I. Global sensitivity indices for nonlinear mathematical models and their Monte Carlo estimated. Mathematics and Computers in Simulation. 2001;55:271–280.

[16] A Saltelli and M Ratto and T Andres and F Campolongo and J Carboni and D Gatelli and M Saisana and S Tarantola. Global sensitivity analysis: the primer. 1st ed. Wiley; 2008.

[17] Wainwright H, Finsterle S, Jung Y, Zhou Q, Birkholzer J. Making sense of global sensitivity analyses. Computers & Geosciences. 2014;65:84–94.

[18] Zhang, X and M Pandey. An effective approximation for variance-based global sensitivity analysis. Reliability Engineering & System Safety. 2014;121:164–174.

[19] Cleveland, W S. Robust locally weighted regression and smoothing scatterplots. Journal of the American Statistical Association. 1979;74(368):819–836.

[20] Cleveland WS, Devlin SJ. Locally-weighted regression: an approach to regression analysis by local fitting. Journal of the American Statistical Association. 1988;83(403):596–610.

[21] Hamilton PT, Richardson JM, Anholt BR. Daphnia in tadpole mesocosms: trophic links and interactions with Batrachochytrium dendrobatidis. Freshwater Biology. 2012;57(4):676–683.

[22] Schmeller DS, Blooi M, Martel A, Garner TW, Fisher MC, Azemar F, et al. Microscopic aquatic predators strongly affect infection dynamics of a globally emerged pathogen. Current Biology. 2014;24(2):176–180.

[23] Buck JC, Truong L, Blaustein AR. Predation by zooplankton on Batrachochytrium dendrobatidis: biological control of the deadly amphibian chytrid fungus? Biodiversity and Conservation. 2011;20(14):3549–3553.

[24] Searle CL, Mendelson JR, Green LE, Duffy MA. Daphnia predation on the amphibian chytrid fungus and its impacts on disease risk in tadpoles. Ecology and Evolution. 2013;3(12):4129–4138.

[25] Grover J, McKee D, Young S, Godfray H, Turchin P. Periodic dynamics in Daphnia populations: biological interactions and external forcing. Ecology. 2000;81(10):2781–2798.

[26] Zippel K, Johnson K, Gagliardo R, Gibson R, McFadden M, Browne R, et al. The Amphibian Ark: a global community for ex situ conservation of amphibians. Herpetological Conservation and Biology. 2011;6(3):340–352.

[27] Becker MH, Harris RN. Cutaneous bacteria of the redback salamander prevent morbidity associated with a lethal disease. PLoS ONE. 2010;5(6):e10957.

[28] Harris RN, Brucker RM, Walke JB, Becker MH, Schwantes CR, Flaherty DC, et al. Skin microbes on frogs prevent morbidity and mortality caused by a lethal skin fungus. The ISME Journal. 2009;3(7):818–824.

[29] Woodhams DC, Vredenburg VT, Simon MA, Billheimer D, Shakhtour B, Shyr Y, et al. Symbiotic bacteria contribute to innate immune defenses of the threatened mountain yellow-legged frog, Rana muscosa. Biological Conservation. 2007;138(3):390–398.

[30] Antwis RE, Preziosi RF, Harrison XA, Garner TW. Amphibian symbiotic bacteria do not show a universal ability to inhibit growth of the global panzootic lineage of Batrachochytrium dendrobatidis. Applied and Environmental Microbiology. 2015;81(11):3706–3711.

[31] Muletz CR, Myers JM, Domangue RJ, Herrick JB, Harris RN. Soil bioaugmentation with amphibian cutaneous bacteria protects amphibian hosts from infection by Batrachochytrium dendrobatidis. Biological Conservation. 2012;152:119–126.

[32] Harris RN, Lauer A, Simon MA, Banning JL, Alford RA. Addition of antifungal skin bacteria to salamanders ameliorates the effects of chytridiomycosis. Diseases of Aquatic Organisms. 2008;83(1):11.

[33] Bletz MC, Loudon AH, Becker MH, Bell SC, Woodhams DC, Minbiole KP, et al. Mitigating amphibian chytridiomycosis with bioaugmentation: characteristics of effective probiotics and strategies for their selection and use. Ecology Letters. 2013;16(6):807–820.
